# Repeated behavioral testing and the use of summary measures reveal trait anxiety in preclinical rodent models

**DOI:** 10.1038/s41398-025-03586-y

**Published:** 2025-10-31

**Authors:** Zoltán K. Varga, Diána Pejtsik, Máté Tóth, Zoltán Balogh, Manó Aliczki, László Szente, Gyula Y. Balla, Levente Kontra, Zsófia Eckert, Huba Szebik, Zsolt Borhegyi, Éva Mikics

**Affiliations:** 1 Translational Behavioural Neuroscience Research Group, HUN-REN Institute of Experimental Medicine, Budapest, Hungary; 2 János Szentágothai Neurosciences Division, Doctoral College, Semmelweis University, Budapest, Hungary; 3 Bioinformatics Core Facility, HUN-REN Institute of Experimental Medicine, Budapest, Hungary

**Keywords:** Neuroscience, Psychiatric disorders

## Abstract

The reliability and validity of preclinical anxiety testing is essential for translating animal research into clinical use. However, the commonly used anxiety tests lack inter-test correlations and face challenges with repeatability. While translational animal research should be able to capture stable individual anxiety traits - the core feature of anxiety disorders - the conventional approach employs a single type of test at a single time, which primarily reflects transient states of animals that are heavily influenced by experimental conditions. Here, we propose a validated, optimized test battery capable of reliably capturing trait anxiety in rats and mice of both sexes. Instead of developing novel tests, we combined widely used tests (elevated plus-maze, open field and light-dark test) to provide instantly applicable adjustments for better predictive validity. We repeated these tests three times to capture behavior across multiple challenges, which we combined to generate summary measures (SuMs). Our approach resolved inter-test correlation issues and provided better predictions for subsequent outcomes under more anxiogenic conditions or fear conditioning. SuMs were also shown to be more sensitive markers of stress-induced anxiety following social isolation. Finally, we tested our method’s efficacy in discovering anxiety-related molecular pathways through RNA sequencing of the medial prefrontal cortex. SuMs revealed four-times more molecular correlates of trait anxiety than transient states, highlighting novel gene clusters. Furthermore, 16% of these correlates were also found in the amygdala. In summary, we provide a novel approach to capture trait anxiety in rodents, offering improved predictions for potential therapeutic targets for personalized medicine. We also provide recommendations to enhance feasibility without compromising validity or animal ethics, tailored to various scientific goals.

## Introduction

Most of us have experienced anxiety during our lives. The tendency to evaluate situations as threatening and respond with anxiety and avoidance stems from one’s individually characteristic, stable trait anxiety (TA) [1, 2]. High levels of TA predict a high frequency of anxious states (SA) in different situations [2–4]. The tendency to experience a heightened level of anxious states represents an important factor in the diagnosis of generalized anxiety disorder (“excessive anxiety and worry […], occurring more days than not for at least 6 months”) [5]. Given its central role in anxiety disorders, TA may represent an important therapeutic target [2, 6, 7].

In pathological conditions of anxiety, pharmacotherapy has a response rate of less than 60% [8], leaving anxiety disorders one of the most prevalent and burdening mental illnesses [9]. Prescribed compounds act through brain mechanisms that have been targeted for at least 30 years with moderate success, and new candidates are rare [10]. Unfortunately, animal models, our primary source of information on the molecular basis of psychiatric disorders, offer targets that fail in clinical trials in more than 90% of cases [11, 12]. Such alarming rates of failed medicine candidates at clinical levels highlights a crisis in our current preclinical approach to modeling psychiatric disorders, including anxiety disorders, and underscores the urgent need for fundamental improvement to enhance translational validity.

One potential reason for this translational gap is the mismatch between what is assessed in preclinical and clinical settings: while clinical anti-anxiety therapies are considered satisfactory if they control the permanent anxiety symptoms, conventionally employed animal tests primarily detect transient and fluctuating anxiety states (SA) [13–15]. Consequently, these tests are more likely to identify markers and treatment targets associated with transient symptoms rather than addressing the underlying drivers of chronic anxious traits. Results from the most commonly used [12] rodent paradigms, the elevated plus-maze (EPM) [16, 17], the light-dark (LD) [18, 19] and the open field (OF) [20, 21] tests, support this concern. Behavioral parameters measured in these tests show limited correlation with each other [13, 22] and are hardly reproducible [23–25], suggesting that in their current use they are unable to capture a common underlying construct like TA. Alternative approaches investigate TA using selectively bred highly anxious animal strains [26–28]. Unfortunately, these approaches are limited to the examination of TA in that specific strain, moreover, their molecular profile may not directly relate to the anxious phenotype. Consequently, despite the clear shortcomings of current methods, and the urgent need for improvement, the field still lacks an adequate tool to measure TA.

**Fig. 1:**
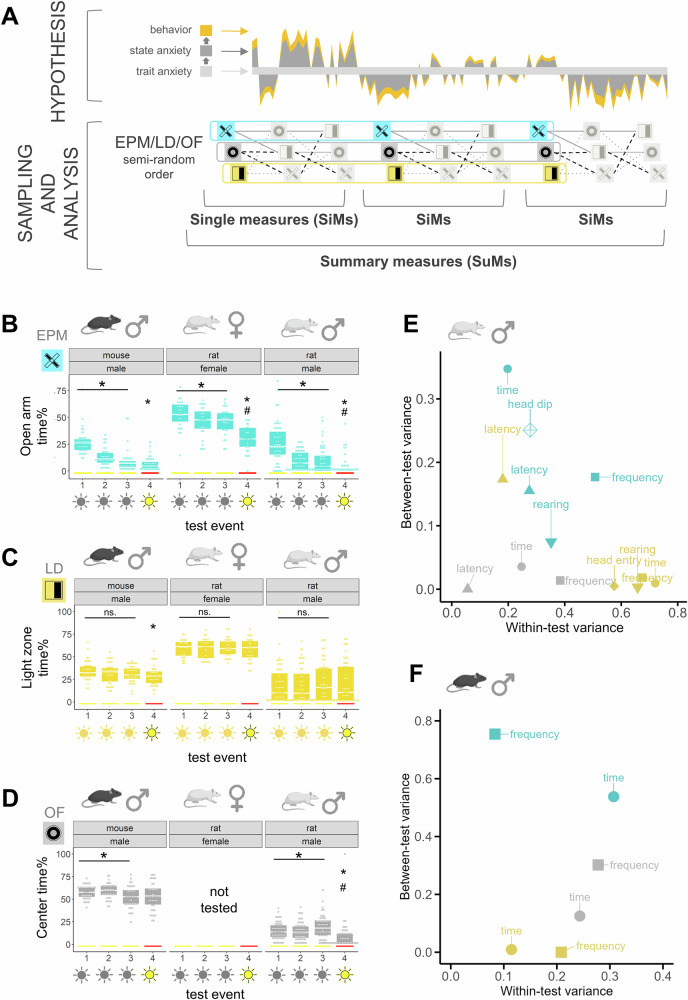
Design and outcome of the extended sampling design of trait anxiety in mice and rats. **A** Hypothesis, sampling, and analysis design. Hypothesis: Schematic drawing showing how behavior is expressed as a consequence of state and trait anxiety according to our working hypothesis. Measured anxiety-like behavior is driven by the underlying trait anxiety, but it is influenced by time and context, hence using a single anxiety test at a single time leads to undersampled experiments reflecting only transient anxiety states of the individual. Sampling design: we used repeated sampling with different types of tests in a semi-random order to better capture internal and external variability stemming from the individual’s trait and state factors, environmental context, and their fluctuation in time. Analysis design: Behavioral data (time or frequency of entering the aversive zone) from each test were used to create classical single measures (SiMs). More stable summary measures (SuMs) were created by combining multiple variables and testing events to describe trait anxiety. **B**–**D** The effect of test repetitions on time spent in the aversive compartment of the EPM (B), LD (C), and OF (D) anxiety tests in male C57BL/6J mice (left panel), female Wistar rats (middle panel) and male Wistar rats (right panel). Red lines highlight the testing event in aversive conditions. The light icons underneath the number of testing occasions represent the type and intensity of light used, where gray means infrared lighting, yellow means white light, and yellow with black contour means aversive lighting condition (low light intensity for the EPM or OF and bright light for LD). Refer to the supplementary figures to see the same plots with aversive zone entry frequencies (Supp. Fig. 1A–C). **E**, **F** Between- and within-test variances of all variables measured for male Wistar rats (E), and male C57BL/6J mice (F). Between-test variance means the individual variance of animals between different test sessions, while within-test variance means the variability of the population’s behavior within a single test session. A higher within-test variance and a lower between-test variance means that a test captures a wide range of individual variation within a test session, and that animals’ behavior is stable across test repetitions. Refer to the supplementary figures for repeatability estimates calculated from between- and within-test variances (Supp. Fig. 1D–E). EPM elevated plus-maze test, OF open field test, LD light-dark test. * over a line: p < 0.05 significant main effect of the test event during the baseline sampling period. * over the aversive sampling day (red line): significant pairwise differences from the 1st test event. # over the aversive sampling day: significant pairwise differences from the 3rd test event. All statistical results are presented in Table 1.

To bridge this gap, we offer a novel approach to measure TA by refining both the sampling and analysis of conventional anxiety tests. We hypothesize that behavior observed in these tests primarily reflect SA, which emerges from the interaction between an individual’s stable TA and the current environmental context (Fig. 1A – hypothesis). Based on this assumption, improved sampling of SA can provide a reliable measurement of TA. Our approach employs the conventional, standard anxiety tests but expands the classical sampling method in both time and depth to capture a broader range of behavioral states (Fig. 1A – sampling and analysis). Specifically, we (i) measured multiple behavioral variables, (ii) included repeated testing sessions and (iii) combined multiple different types of tests in our analysis. Particularly, we sampled SA in rats and mice using different sequences of the most widely used anxiety tests, with each test repeated three times for all animals. We measured a range of variables (referred to as single measures, SiMs) and assessed the possibility of their summarization. This led to the creation of summary measures (SuMs) by averaging SiMs across repeated tests, and composite measures (COMPs) by averaging SiMs or SuMs across different test types (Fig. 1A). In medical research, generating SuMs from serial sampling is a well-established strategy for capturing the underlying, stable construct of fluctuating states while reducing environmental noise and avoiding over-parametrisation [29]. Despite these clear advantages, this approach has yet to be adopted by the field of behavioral neuroscience.

We validated SuMs as indicators of TA by several novel approaches. (i) Since TA is assumed to underlie performance across different anxiety tests, we hypothesized that SuM-based inter-test correlations would be stronger than the classical SiM-based ones. (ii) Since TA is stable across time and contexts, we hypothesized that SuMs better predict future behavioral responses to acute stress in anxiety- or fear-triggering environments. (iii) Since high levels of TA is a core symptom of anxiety disorders, we further hypothesized that SuMs would serve as more sensitive markers of chronic stress-induced anxiety in an etiological stress model. Following these validation steps, we examined the sensitivity of SuMs in discovering molecular markers and potential therapeutic candidates for anxiety by conducting RNA sequencing in the medial prefrontal cortex (mPFC), a key anxiety-related brain region [30, 31].

## Materials and methods

Detailed methods and a table outlining the specific procedures applied to each cohort are provided in the supplementary materials (Supp. Mat. Table 1).

### Animals

Six animal cohorts were used in this study, each acclimated to the housing conditions for two weeks before testing. Animals were housed in groups of 2–5 (mice and rats in separate rooms) under standard conditions with a reversed day-night cycle. The cohorts included: two cohorts of adult male Wistar rats (n = 54, Charles-River) to examine trait anxiety and neural correlates; one cohort each of adult female Wistar rats (n = 27), adult male C57BL/6J mice (n = 40, Jackson Laboratory) to assess replicability and sex differences; adult male Long-Evans rats (n = 28, Charles-River) to study correlations between acoustic startle, generalized fear (CFP), and trait anxiety; and adult male Wistar rats (n = 30) to investigate effects of social isolation on trait anxiety. A table summarising all cohorts and tests can be found in the supplementary methods (Supp. Mat. Table 1). Sample sizes for the first rat experiment (n = 54) were calculated using the pwr R package aiming to measure a minimum r = 0.35 correlation coefficient at alpha = 0.05 significance level and beta = 0.8 power. Following the first experiment we decreased sample sizes according to the robustness indices of behavior-behavior and behavior-gene expression correlations.

### Experimental design

Wistar rats underwent a semi-randomized 3-week test battery (EPM, OF, LD tests, each repeated three times), with six fixed test order combinations to eliminate order and time-of-day biases (Fig. 1A). This was followed by an additional week under more aversive conditions (increased light), and, two days later, a final open field test (OF2) to standardize the last testing experience for all animals. For the social isolation protocol, rats were reared from weaning (P21) either in groups of four or isolated until adulthood. Female rats, socially isolated males, and mice underwent the same protocol of 3-week baseline testing followed by an aversive testing week. Long-Evans rats completed the 3-week protocol, followed by an acoustic startle and fear conditioning tests. For anxiety tests, time spent in the aversive zone, frequency, and latency of aversive zone entries were analyzed automatically (Noldus EthoVision XT15), while EPM head-dips, LD head-entries, and rearing were scored manually (Solomon Coder [32]) by experimenters blind to experimental groups.

### Behavioral measures

**Fig. 2:**
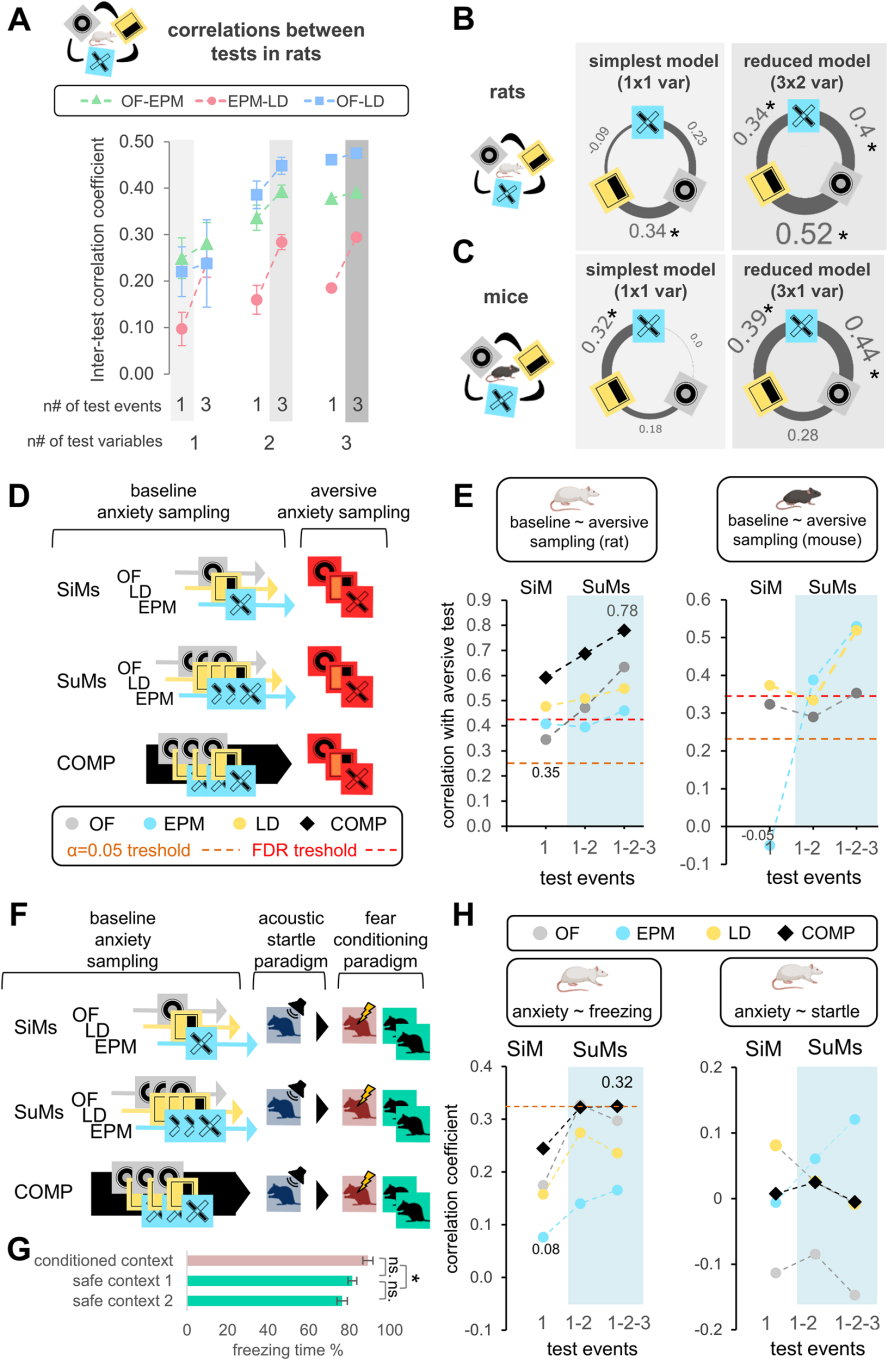
Inter-test correlations and behavioral predictions as a function of variable complexity. **A** Inter-test correlations between increasingly complex variables of different anxiety tests. The correlation coefficients between OF and EPM (green), EPM and LD (red), and LD and OF (blue) were plotted at different variable combinations of these tests. The #n of test events and the #n of test variables refers to how many repeated test events (1 or 3 repeats) were included and the number of different variable types (time, frequency, latency) that were used to create summary variables, respectively. SiMs are single variables, for example time spent in the open arms of an EPM, while SuMs consist of either multiple samplings of one variable type and/or multiple variable types measured in one or multiple events/repeats. **B**, **C** Network plot of inter-test correlations between different tests revealed by SiMs (simplest model) and SuMs (reduced) in the rat (B) and mouse (C) experiments. * p < 0.05 significant correlation **D** Behavioral predictions of anxiety measures in an aversive environment. Correlation coefficients were measured between identically composed variables in baseline and aversive contexts. SiMs represent one, and SuMs represent multiple testing events, while COMPs include SiMs or SuMs of all test types. **E** Correlations between increasingly complex anxiety measures (SiMs, SuMs, or COMPs) from the baseline sampling and their aversive counterparts in rats (left) and mice (right). **F** Behavioral predictions of anxiety measures in the fear conditioning paradigm. Correlation coefficients were measured between baseline anxiety measures (SiMs, SuMs, COMPs), freezing in the fear conditioning paradigm, and startle in the acoustic startle paradigm. **G** Time spent with freezing (%) in the conditioned fear paradigm in the contextual reminder (conditioned) and safe (different) contexts. Freezing was sampled two times in the safe context, from which an average freezing value was used for predictions. **H** Correlation of increasingly complex anxiety measures (SiMs, SuMs, or COMPs) with freezing behavior in a novel context following fear conditioning (left) and startle response to acoustic stimuli (right). COMP represents the composite of all anxiety tests and measures. Red dashed lines represent the threshold of significance on the scale of correlation coefficients. Additional numbers on the plots show the smallest and largest coefficients. Figures A-E show male Wistar rats and male C57BL/6J mice, while Figures F-H display results from experiments with male Long-Evans rats. All statistical results are presented in Fig. 5A). Statistical parameters of Fig. 2G) can be found in Table 1.

Composition of all variables are shown below. To attenuate the possibly state anxiety-driven fluctuation of behavioral variables across repeated testing, and thus to improve the detection of stable traits, we introduced summary measures of behavior. Our improvement to the conventional preclinical approach involved two key aspects: (1) Based on the results of the principal component analysis shown in Supp. Fig. 1F–G, and correlations shown in Fig. 2A, B as well as Supp. Fig. 2A, we calculated single measures (SiMs) by inverting min-max-scaled time spent in the aversive zone (for rats) or frequency of entering the aversive zone (for mice) during the first exposure to each test (EPM, OF, LD performed during the first week of testing). (2) Using data from the same animals, we also created summary measures (SuMs) by averaging and summing the scaled variables across the 2-, or 3 repetitions of each anxiety test type. This approach aimed to reduce noise from situational variability and enhance the detection of temporally consistent traits. To assess the effectiveness of SuMs in capturing the common underlying factors across different anxiety tests, we compared the strength of individual-level correlations using SiMs versus SuMs. Additionally, we also created composite scores (COMP) by averaging the SiMs or SuMs of different tests. Means and standard errors are reported for behavioral measures.

Throughout this study, all variables were scaled into dimensionless, combinable values ranging from 0 to 1 (min-max scaling) and inverted (multiplied by −1) to correspond to anxiety levels according to the following formula:

$${scaled\; variable}(i)=\left(\frac{{variable}(i)-\min \left({variable}\right)}{\max \left({variable}\right)-\min \left({variable}\right)}\right)* (-1)$$

SiMs, SuMs or COMPs were calculated using the following scaled and inverted variables:

**Calculation of single measures for a test (SiMs):**

***rats: SiM*** *= time spent in aversive zone*

***mice: SiM*** *= entry frequency into aversive zone*

**Calculation of summary measures (SuMs):**

***rats: SuM*** *of 3 tests of the same test type = average(****test1****[time+freq],* ***test2****[time+freq],* ***test3****[time+freq])*

***mice: SuM*** *of 3 tests of the same test type = average(****test1****[freq],* ***test2****[freq],* ***test3****[freq])*

**Calculation of composite scores (COMPs) of different test types:**

***rats and mice: COMP SiMs*** *of different test types = average(****LD****[time+freq],* ***EPM****[time+freq],* ***OF****[time+freq])*

***rats and mice: COMP of SuMs*** *of different test types = average(****LD SuM****[time+freq],* ***EPM SuM****[time+freq],* ***OF SuM****[time+freq])*

**Fig. 3:**
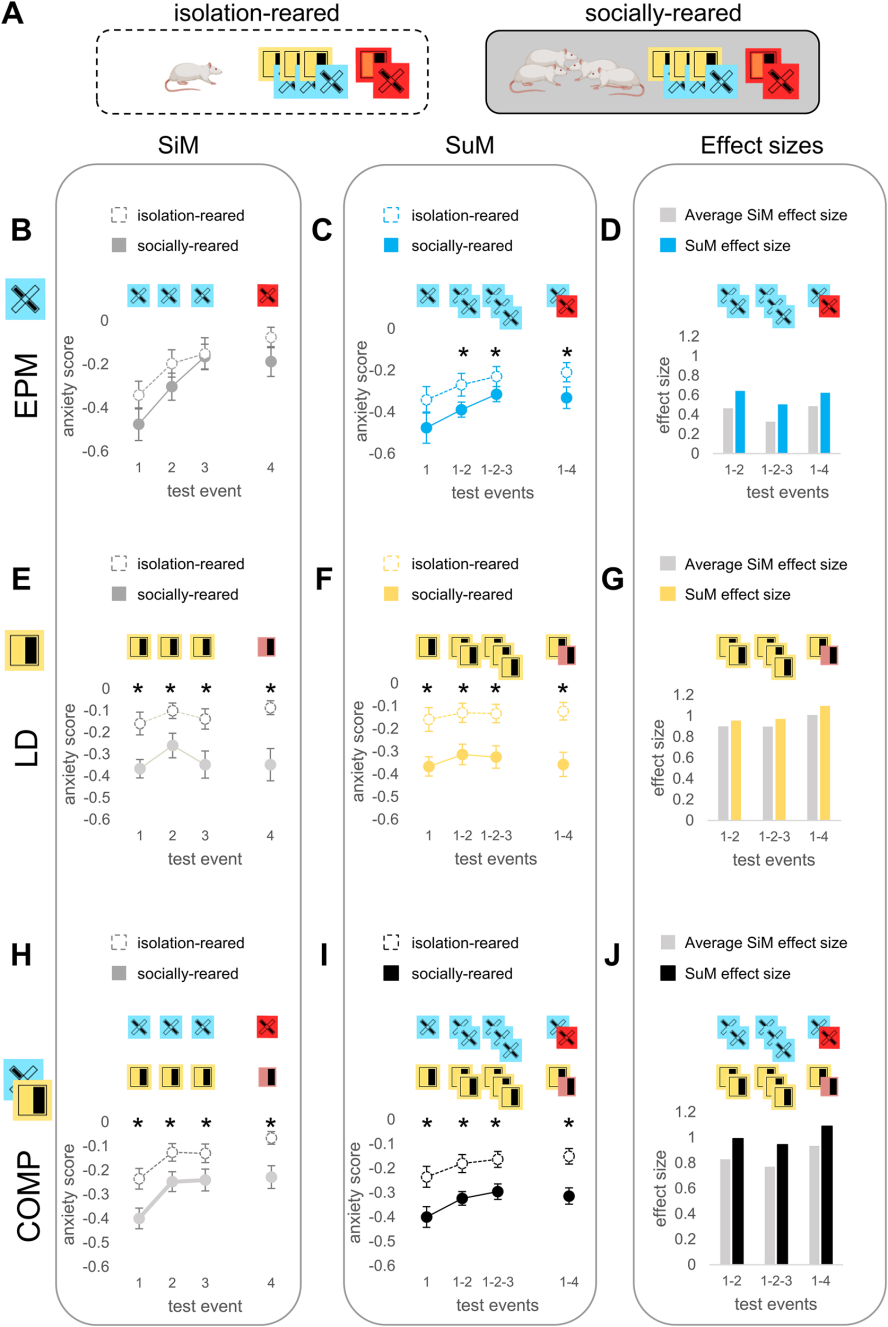
Summary measures as markers of a chronic stress state. **A** Socially or isolation-reared animals underwent three baseline and one aversive sampling events of the EPM and LD tests, then summary measures of different complexities were calculated. **B**-**E**-**H** SiMs (scaled and inverted time and frequency-based anxiety scores) were calculated from each sampling event using the EPM (B) or LD tests (E), or their composite score (COMP) (H). **C-F-I** SiMs (1 test event) were calculated from the first, while SuMs were calculated from multiple averaged sampling events (1-2, 1-2-3, 1-4), using the EPM (C), LD (F), or their composites (I). **D**-**G**-**J** The effect sizes of isolation rearing based on averaged sampling events (SuMs) compared to the averages of effect sizes of those very same sampling events without summarization. Gray and colored bars represent the effect sizes of the comparisons from the first (B-E-H) and the second columns (C-F-I), respectively. Experiments in this figure were done using male Wistar rats. All statistical results are presented in Table 1. * significant difference after FDR correction.

Anxiety scores used in Fig. 3 are SiMs or SuMs containing scaled and inverted time and frequency data. An additional table (Supp. Mat. Table 2) with variable components in the Supplementary methods is available.

**Statistical analysis** was done in R statistical environment [33]. Principal component analysis [34] of all analyzed behavioral variables from the first week of testing was performed to investigate the shared variance between different behavioral variables. For correlations, Spearman correlation analysis [35] was used. False discovery rate (FDR) correction for multiple comparison testing was done using the Benjamini-Hochberg method [36].

**Repeatability** was assessed by calculating the proportion of within-test variance to total variance [37] using the formula: *within test variance/(within test + between test variance)*. Within-test variance reflects individual variation within a single test, while between-test variance captures individual variation across test repetitions. Variance components and repeatability estimates (with 95% confidence intervals via 1000 parametric bootstraps) were computed using ‘rptR’ package [38, 39]. Estimates were deemed significant if confidence intervals excluded zero. After verifying normality, variances and adjusted repeatability values were determined for all behavioral measures.

**Random forest analysis (RF)** with automated variable selection was performed using the VSURF package [40] in R. Subjects were assigned to low or high anxiety groups according to whether their anxiety score fell below or above the interquartile density minimum (valley). As a result of RF and the VSURF variable selection pipeline a list of significant predictors was chosen to maximize variable importance and minimize redundancy. Variable importance scores of predictors were plotted against their respective variable complexity.

### Blood corticosterone assessment of Wistar rats

Tail vein blood samples were collected in a resting state (5 days before the first experiment) and in stress-induced (immediately after OF2) conditions. The quantification of plasma corticosterone was carried out using radioimmunoassay similarly to previous work by our laboratory [41].

### RNASeq analysis

**Fig. 4:**
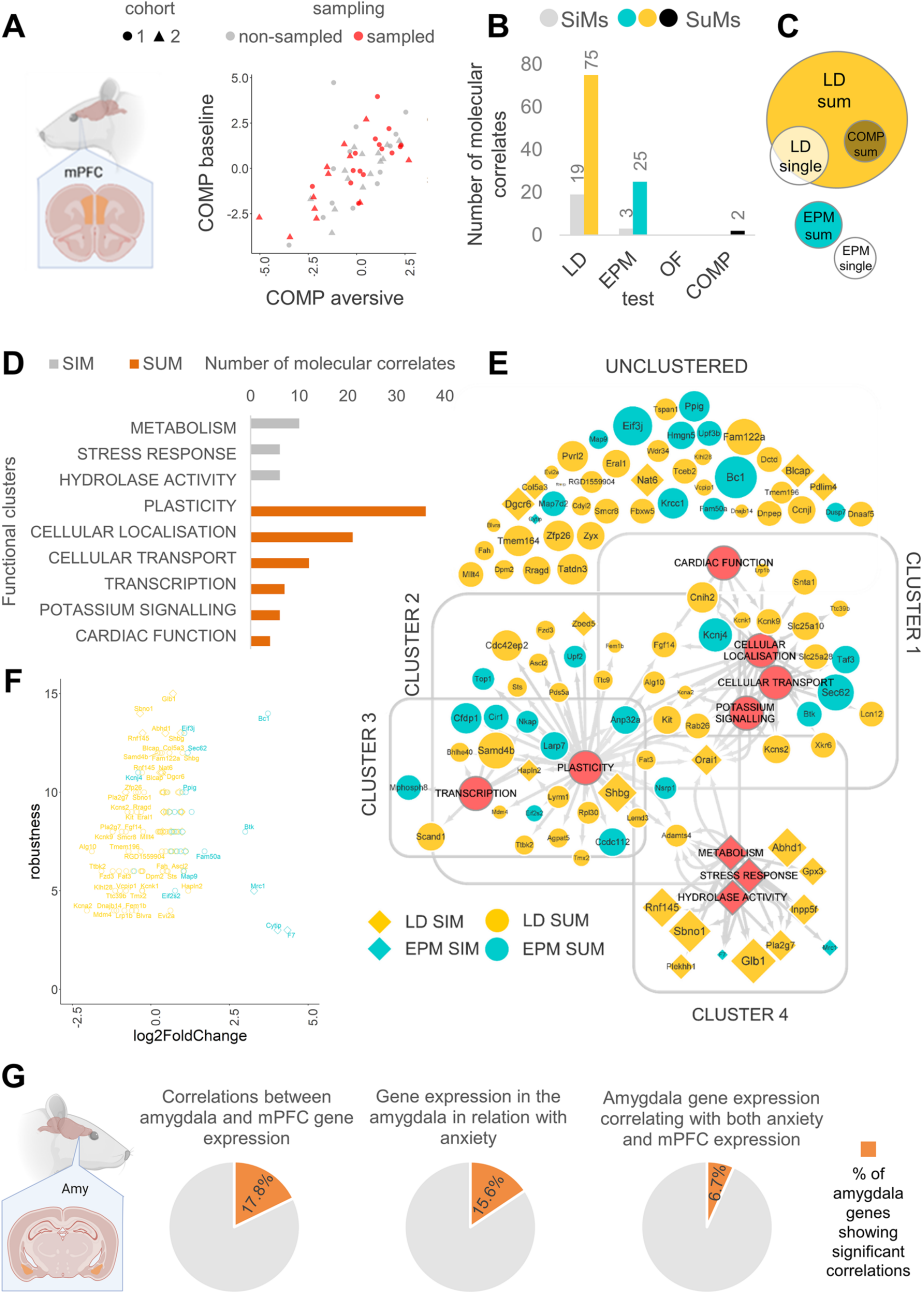
Sensitivity to gene-discovery of differently complex anxiety measures. **A** left: Representative figure of bilateral mPFC sampling (orange areas). right: Samples for RNA-seq analysis were chosen based on their COMP levels. **B** SiM and SuM-associated gene counts following FDR correction for multiple comparisons. **C** Euler diagram representing the proportion of overlaps in gene sets of different sampling approaches. **D** SiM and SuM-associated genes form functionally distinct clusters. Clusters contain genes with similar annotation profiles based on multiple databases. SiM and SuM-associated genes are grouped into completely non-overlapping clusters defined by metabolism or plasticity, respectively. **E** Hub-plot of functional annotation of significant genes. Due to the heuristic fuzzy clustering method, a gene does not have to belong to any clusters but can belong to more clusters, while clusters can be determined by multiple functional labels. Cluster 1 includes genes that share many functional labels associated with cardiac function, cellular localization, transport, and potassium signalling. Clusters 2 and 3 are solely characterized by plasticity and transcription functions, respectively. Cluster 4 is characterized by metabolism, stress response, and hydrolase activity-associated functional terms and unites almost exclusively SiM-associated genes. Unclustered genes do not necessarily lack a known function, but do not have the amount of relatedness with enough genes in our pool to form a separate cluster. Gray arrows indicate gene-function associations, while gray lines indicate the borders of clusters. Red circles indicate cluster-determining labels, while color and shape indicate sampling type. The size of hubs is proportional to the correlation coefficient (absolute value) between the gene expression and anxiety. **F** Robustness values of genes as a function of their log2-fold change. **G** Correlations of amygdala gene expressions with mPFC gene expression or anxiety variables. Left piechart: percent of significant correlations between amygdala and mPFC qPCR results. middle: percent of significant correlations between amygdala gene expression and SiMs, SuMs, COMPs or aversive behavior. right: percent of amygdala genes that correlate with both anxiety and mPFC expression. Experiments in this figure were done with male Wistar rats. All statistical results are presented in Table 1 and Supplementary Tables 1–3.

Microdissection of bilateral mPFC tissue was performed 2 weeks after the last experiment (OF2). Whole transcriptome-analysis via RNASeq was performed on the homogenized mPFC tissue. Single-read RNAseq was performed on 27 animals, chosen based on their COMP scores (Fig. 4A), by the Department of Biochemistry and Molecular Biology at University of Debrecen, using the NEBNext Ultra II RNA Library Prep Kit (New England Biolabs) and sequencing on Illumina NextSeq500. Regression analysis to reveal differentially expressed genes (DEGs) was done using the DESeq2 package [42]. Analysis of gene-behavior regressions was carried out using SiMs, SuMs or COMPs of tests as continuous covariates in the design. FDR correction was done using the Independent Hypothesis Weighting method [36]. Only genes with FDR-adjusted p < 0.05 were considered significant. Functional clustering of DEGs was done using DAVID [43, 44], and connections were visualized in Cytoscape [45]. Deseq2 results are shown in Supplementary Table 1.

**qPCR analysis** was performed on total RNA from mPFC and amygdala of the same animals as RNASeq. Gene expression was measured using custom TaqMan Array Cards (Applied Biosystems, USA) for 45 genes. Inter-run calibration used Actb and Gapdh as references, and data were normalized using the 2-ΔΔCT method [46, 47]. Expression values with cycle thresholds >35 were excluded.

**Robustness**. We assessed the stability of correlations between behaviors and gene expression via a robustness index, based on the minimum sub-cohort size where the standard deviation of p-values reached the 0.05 threshold. This was calculated by reanalyzing Spearman correlations across 20,000 random sub-cohorts; the R function is available upon request.

## Results

### Overlaps between anxiety-like variables support the use of SuMs

When variables carry overlapping information, summarizing them can be both feasible and beneficial for capturing their shared background. To assess the level of overlap among variables from the EPM, LD, and OF tests and to measure behavioral changes across repeated test sessions, we conducted repeatability and correlation analyses in both rats and mice. Repeatability was assessed by examining test-retest changes across repeated sessions under baseline and more aversive conditions (bright light) (Fig. 1B–D), as well as through within and between-test variability (Fig. 1E, F), and bootstrap analysis-based repeatability measures (Supp. Fig. 1D, E). During repeated testing in baseline conditions, a significant change in time spent in the aversive zone of the tests was observed over repeated sessions in the EPM (Fig. 1B) and the OF (Fig. 1D) in both rats and mice, but not in the LD test (Fig. 1C). Testing under more aversive conditions tended to decrease time spent in the aversive zone in the EPM and OF, but not in the LD test. Aversive zone entries showed similar trends, with a more pronounced avoidance response to the aversive light conditions, and some effects of repeated testing in the LD (Supp. Fig. 1A–C). The observed test-type-dependent effects demonstrate that different anxiety tests vary in their sensitivity to environmental influences. Notably, the LD test showed minimal test-retest carry-over or aversive condition effects, while the EPM and the OF tests demonstrated high sensitivity to both factors. Analysis of within- and between test variances, and test repeatability (Fig. 1E, F, Supp. Fig. 1D, E) confirmed that the LD variables are the most stable across test repetitions (lowest individual variability between test sessions) in rats and mice, and the most sensitive to individual differences (highest population variability within a test session) in rats, therefore the LD had the highest repeatability values for most variables (frequency and time spent in aversive zone, head entries into aversive zone and rearing). In contrast, the EPM showed the highest variance across repeated testing in both rats and mice, and showed the lowest repeatability for most variables (Fig. 1E, F, Supp. Fig. 1D, E). Variables of the same test type clustered together based on their between- and within-test variances, with frequency variables showing the highest, and latency-variables the lowest repeatability in rats, indicating a partial overlap among variables measured in the LD, EPM or OF. PCA revealed further similarities, as variables of all three tests tended to load onto the same dimensions (Supp. Fig. 1F–G). Since inclusion of ethological parameters, such as head-dipping or rearing showed no clear PCA separation or superior repeatability compared to time or frequency measures, we prioritized these simpler, robust readouts in later analyses. Interestingly, while in rats time and frequency variables of different tests loaded onto PC1 with similar weight, in mice only frequency variables were represented consistently in the most important component. Statistical results for (Fig. 1B−D, and Supp. Fig 1A−C) are presented in Table 1.

**Table 1:** Parameters of test statistics.

Figure / result	Hypotheses testing / model	comp.	var.	Test statistics with df	n	effect size	p	Further information
Figure 1B/C/D	repeated measures ANOVA / rat male main effect	EPM	time(s)	F2,107 = 34.206	54	0.800	0.000	
			freq	F2,107 = 26.517		0.703	0.000	Supp. Fig. 1A
		OF	time(s)	F2,108 = 3.643		0.260	0.029	
			freq	F2,108 = 2.235		0.204	0.112	Supp. Fig. 1C
		LD	time(s)	F2,106 = 0.801		0.123	0.452	
			freq	F2,106 = 4.119		0.279	0.019	Supp. Fig. 1B
	aversive contrast paired t-test (male rats)	EPM 1 vs 4 (aversive)	time(s)	t76 = 8.007		0.920	0.000	
			freq	t82 = 6.738		0.742	0.000	Supp. Fig. 1A
		EPM 3 vs 4 (aversive)	time(s)	t94 = 2.898		0.299	0.005	
			freq	t94 = 2.748		0.283	0.007	Supp. Fig. 1A
		OF 1 vs 4 (aversive)	time(s)	t88 = 2.894		0.309	0.005	
			freq	t106 = 4.643		0.451	0.000	Supp. Fig. 1C
		OF 3 vs 4 (aversive)	time(s)	t102 = 4.043		0.400	0.000	
			freq	t104 = 4.9		0.479	0.000	Supp. Fig. 1C
		LD 1 vs 4 (aversive)	time(s)	t106 = −0.315		0.031	0.753	
			freq	t107 = −0.460		0.045	0.646	Supp. Fig. 1B
		LD 3 vs 4 (aversive)	time(s)	t106 = 0.356		0.035	0.722	
			freq	t105 = 1.078		0.105	0.284	Supp. Fig. 1B
	repeated measures ANOVA / rat female main effect	EPM	time(s)	F2,52 = 3.419	27	0.363	0.040	
			freq	F2,52 = 10.343		0.631	0.000	Supp. Fig. 1A
		LD	time(s)	F2,52 = 0.038		0.038	0.963	
			freq	F2,52 = 16.647		0.800	0.000	Supp. Fig. 1B
	aversive contrast paired t-test (female rats)	EPM 1 vs 4 (aversive)	time(s)	t51 = 7.989		0.853	0.000	
			freq	t49 = 0.595		1.121	0.000	Supp. Fig. 1A
		EPM 3 vs 4 (aversive)	time(s)	t52 = 4.431		0.616	0.000	
			freq	t51 = 3.609		0.506	0.001	Supp. Fig. 1A
		LD 1 vs 4 (aversive)	time(s)	t50 = 0.594		0.085	0.555	
			freq	t52 = 1.712		0.243	0.093	Supp. Fig. 1B
		LD 3 vs 4 (aversive)	time(s)	t50 = 0.675		0.096	0.503	
			freq	t52 = −2.073		0.289	0.043	Supp. Fig. 1B
	repeated measures ANOVA / mouse main effect	EPM	time(s)	F2,78 = 64.944	40	1.290	0.000	
			freq	F2,78 = 74.446		1.382	0.000	Supp. Fig. 1A
		OF	time(s)	F2,77 = 8.386		0.466	0.001	
			freq	F2,77 = 23.279		0.779	0.000	Supp. Fig. 1C
		LD	time(s)	F2,78 = 1.411		0.190	0.250	
			freq	F2,77 = 0.971		0.159	0.383	Supp. Fig. 1B
	aversive contrast paired t-test (mouse)	EPM 1 vs 4 (aversive)	time(s)	t59 = 9.806		1.272	0.000	
			freq	t77 = 12.936		1.478	0.000	Supp. Fig. 1A
		EPM 3 vs 4 (aversive)	time(s)	t62 = 1.192		0.152	0.238	
			freq	t78 = 1.139		0.129	0.258	Supp. Fig. 1A
		OF 1 vs 4 (aversive)	time(s)	t59 = 1.774		0.230	0.081	
			freq	t54 = 9.281		1.265	0.000	Supp. Fig. 1C
		OF 3 vs 4 (aversive)	time(s)	t74 = −0.289		0.034	0.773	
			freq	t71 = 5.077		0.604	0.000	Supp. Fig. 1C
		LD 1 vs 4 (aversive)	time(s)	t78 = 2.468		0.280	0.016	
			freq	t76 = 3.0342		0.349	0.003	Supp. Fig. 1B
		LD 3 vs 4 (aversive)	time(s)	t76 = 1.054		0.121	0.295	
			freq	t78 = 1.6831		0.191	0.096	Supp. Fig. 1B
Figure 2A	Linear-mixed model’s	complexity	corr coeff	t = 3.597; Est = 0.019; SE = 0.005	54	-	0.001	variable composition: p = 0.0065
								test composition: p = 0.0088
								Residual: 0.0042
Figure 2G	ANOVA	freezing	time(s)	F2,25 = 7.000	28	0.748	0.001	
	Tukey post-hoc	conditioned context (ctx) vs safe ctx1		t135 = 2.247; Est = 7.69; SE = 3.42		0.390	0.067	
		cond ctx vs safe ctx2		t135 = 3.715; Est = 12.71; SE = 3.42		0.640	0.001	
		safe ctx1 vs safe ctx2		t135 = 1.468; Est = 5.02; SE = 3.42		0.250	0.309	
Figure 3B	Wilcoxon rank sum test	EPM 1	anxiety score	w = 86	30	0.490	0.143	FDR: non-significant
		EPM 2		w = 74		0.440	0.056	
		EPM 3		w = 97		0.050	0.263	
		EPM 4(aversive)		w = 34		0.480	0.023	
Figure 3C	Wilcoxon rank sum test	EPM 1-2		w = 37		0.640	0.023	FDR: all significant
		EPM 1-2-3		w = 36		0.500	0.034	
		EPM 1-4(aversive)		w = 34		0.620	0.020	
Figure 3E	Wilcoxon rank sum test	LD 1		w = 41		0.990	0.001	FDR: all significant
		LD 2		w = 50		0.810	0.004	
		LD 3		w = 42		0.890	0.001	
		LD 4 (aversive)		w = 43		1.030	0.002	
Figure 3F	Wilcoxon rank sum test	LD 1-2		w = 38		0.960	0.001	FDR: all significant
		LD 1-2-3		w = 43		0.970	0.002	
		LD 1-4(aversive)		w = 33		1.100	0.000	
Figure 3H	Wilcoxon rank sum test	COMP 1		w = 52		0.900	0.006	FDR: all significant
		COMP 2		w = 52		0.756	0.006	
		COMP 3		w = 64		0.653	0.023	
		COMP 4(aversive)		w = 39		0.969	0.001	
Figure 3I	Wilcoxon rank sum test	COMP 1-2		w = 37		0.992	0.001	FDR: all significant
		COMP 1-2-3		w = 36		0.944	0.000	
		COMP 1-4(aversive)		w = 34		1.089	0.000	
Supp. Fig. 3A	Wilcoxon rank sum test	EPM baseline vs aversive	anxiety score	w = 1512.5	54	0.374	0.006	
		OF baseline vs aversive		w = 1813		0.659	0.000	
		LD baseline vs aversive		w = 1229		0.076	0.575	
Supp. Fig. 3B	ANOVA	corticosterone	concentration	F = 44.225	28	1.844	0.000	
	Tukey post-hoc	aversive vs resting 1		t81 = 8.407; Est = 426; SE = 50.6		1.870	0.000	
		aversive vs resting 2		t81 = 7.854; Est = 398; SE = 50.6		1.750	0.000	
		Resting 1 vs resting 2		t81 = −0.554; Est = −28; SE = 50.6		0.120	0.845	

Table 1 shows (left-to-right) the result section/figure number corresponding to the statistical parameters, the hypotheses testing method/model that were used, the group comparisons that were made, the variables that were assessed, and the resulting test statistics-values with degrees of freedom, sample sizes, effect sizes and significance levels. We presented the conventional parameters of the particular statistical approaches such as F values in the case of ANOVAs, t or w values in the case of t-tests or Wilcoxon tests, as well as estimates (est) and standard errors (SE) in the case of linear mixed models. Numbers following the test statistics identifier (e.g. F or t) are the within and between group degrees of freedom values, separated by a comma. Effect sizes are Cohen’s f for ANOVA and Cohens’s d for t-tests. In the case of Wilcoxon test, we calculated effect sizes as the difference between the means of compared groups, divided by the standard deviation of those two groups. P values of 0.000 represent values below 0.0005. The column entitled Further information shows additional relevant information that is specific for a particular method, like random variances in mixed models or the results of FDR correction following multiple comparisons.

In summary, our results indicate a shared but differently expressed underlying basis in these anxiety tests and support the potential for summarizing their variables.

### SuMs reveal shared variance across anxiety tests and predict acute stress-induced behavior

Since TA is considered to be the permanent basis of anxiety-like responses [48, 49], it should be consistently expressed across different contexts and over time. To test this, we created SuMs of varying complexity through averaging time and frequency variables of repeated test results from the EPM, LD, and OF tests. We then assessed (i) correlations between SuMs and their predictive efficacy for behavior in different stress-inducing contexts, including (ii) performance in the same tests under more aversive conditions (Fig. 2D, E), (iii) fear responses in the conditioned fear paradigm (Fig. 2F–H, Table 1) or (iv) acoustic startle paradigms (Fig. 2H). We also conducted random forest classification to compare SiMs and SuMs in predicting behavior in the aversive context and the fear conditioning paradigm.

The simplest variables (SiMs) were the time spent in, frequency and latency to enter the aversive compartment. The most complex SuMs consisted of all these variables across three testing sessions. We generated all variable combinations along this complexity gradient and assigned each a complexity score, calculated as the number of variables * number of test sessions (Supp. Fig. 2A).

**Fig. 5:**
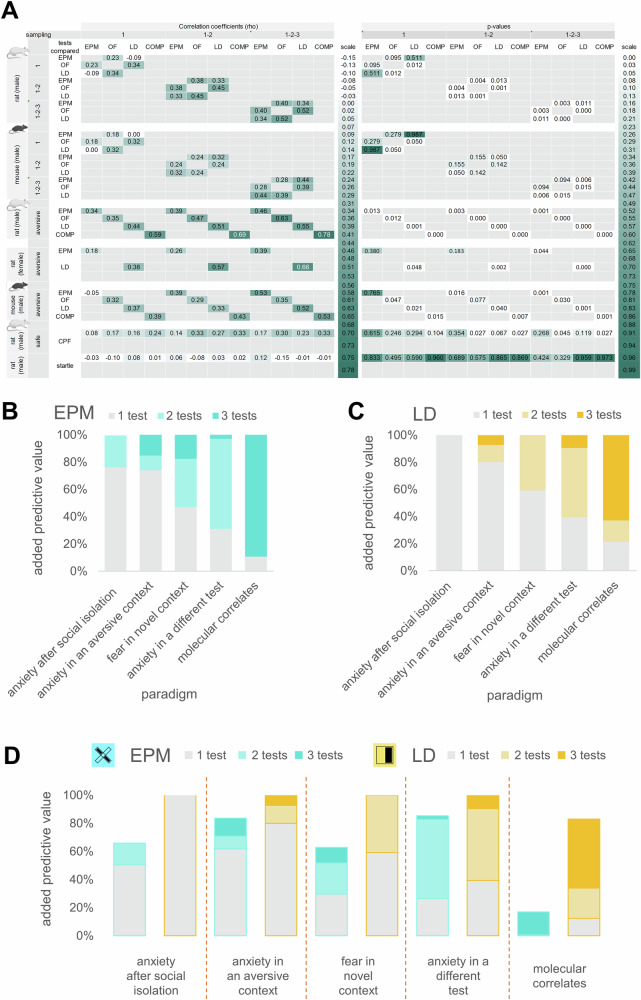
Predictive value of the extended sampling design in different paradigms. **A** Correlation coefficients and corresponding p-values of the “inter-test correlations” and “behavioral predictions” experiments in rats and mice. The color code of the cells ranging from white to teal represents the level of correlations or p-values scaled to all parameters and experiments. The last two rows of results (conditioned fear paradigm and startle) are from experiments with male Long-Evans rats, and the rest is with male or female Wistar rats or male C57BL/6J mice, sex and species are marked in the table. **B**, **C** The advantage of multiple testing in different paradigms. Additional predictive value of further sampling points in different paradigms. Values are shown as the percentage of the highest prediction that was reached with a particular test in a particular paradigm. **D** Additional predictive values are shown as a percentage of the highest prediction that was reached with any of the tests used in a particular paradigm.

We found that SuMs of high complexity showed stronger inter-test correlations than either lower-complexity SuMs or SiMs, in both rats and mice (Figs. 2A–C, 5A). Inter-test correlations could be explained by variable complexity as a fixed effect, and variable and test composition as interacting random effects (Supp. Fig. 2A). The weakest correlations were observed for the least complex variables, and SuMs that included latency data, while the strongest correlations were between SuMs composed of more complex metrics, and/or the ones that included frequency and time-based variables (Supp. Fig. 2A). Based on these results and the previous PCA (Supp. Fig. 1F–G), we used time and frequency but not latency variables in our reduced SuMs in rats (anxiety scores), which yielded stronger inter-test correlations and outperformed SiMs. Similarly, in mice, the strongest inter-test correlations were between PC1-loaded variables, but in contrast to rats, these were the frequencies. Based on these, we used frequency variables both in SiMs and SuMs in mice in all following analyses. This species-specific approach optimizes cross-study comparisons while respecting each model’s unique behavioral profiling characteristics.

Under more aversive conditions, we detected enhanced anxiety-like behavior in two out of the three tests in rats and in all tests in mice (time: Fig. 1B–D, frequency: Supp. Fig. 1A–C, anxiety score: Supp. Fig. 3A). Correlation analysis between test-specific SiMs and SuMs and their aversive-condition counterparts revealed that more complex variables better predicted future behavioral responses in both species (Fig. 2E). Furthermore, creating composite scores from SiMs and SuMs of all tests (COMP) outperformed test-specific SiMs and SuMs, respectively, reaching a 0.78 correlation between baseline and aversive responses.

We extended the above analyses to both species and sexes, and found a similar association between the number of sampling events and their predictive power (Fig. 5A). We also confirmed the importance of variable complexity in predicting behavior by performing random forest classification (RF) of low- and high-anxiety subjects in aversive condition (Supp. Fig. 3C–E). Subjects were grouped based on their anxiety scores under aversive testing for each test type, and RF was conducted using variable combinations from previous tests. The strongest predictors of anxiety scores aversive LD, OF, and EPM tests were: SuMs of the first two LD tests (anxiety score), the first three OF tests (time), and the first two EPM tests (anxiety score), respectively. Furthermore, we observed a strong positive correlation between variable complexity and variable importance as determined by the random forest model (Supp. Fig. 3F). Note that correlations with significant SuM predictors were higher than the correlations with most of our SiMs (1st, 2nd, or 3rd testing) indicating that their predictive performance is not merely the result of carry-over effect of repeated testing, but rather reflects the capture of additional meaningful information (Supp. Fig. 3G, H). Likewise, the predictions of COMPs were higher than the predictions of SuMs, which indicates that they carry additional information compared to any test-type alone (Supp. Fig. 3G, H). Furthermore, inter-test correlations of SiMs were also independent of testing occasion in contrast to SuMs with correlations consistently growing by variable complexity (Supp. Fig. 2B).

Similarly to our previous studies, exposure to an anxiety test induced a rapid increase in plasma corticosterone levels (Supp. Fig. 3B, Table 1) but neither baseline nor stress-induced corticosterone correlated with anxiety variables [50] (data not shown).

We also aimed to predict the extent of freezing behavior in a safe context in the conditioned fear paradigm as a measure of generalized fear, a hallmark symptom of post-traumatic stress disorder [51]. While SiMs of anxiety tests showed poor performance in predicting fear responses, we observed a notable improvement when more complex variables were used, especially COMPs (Figs. 2H, 5A). In contrast, acoustic startle responses were not predictable by any variable or sampling method indicating a specificity of SuMs. Random forest classification of low and high freezing animals (Supp. Fig. 3I) indicated the LD test as the best predictor of fear generalization (Supp. Fig. 3K), however the model showed weak accuracy (Supp. Fig. 3J). These results indicate that SuMs and their composite measures can increase predictions of specific future behavioral states in challenging/stressful environments.

#### SuMs are more sensitive markers of chronic stress-induced behavior

Since TA is an important risk factor for the development and maintenance of anxiety disorders [2], we expected TA markers to be sensitive to etiological models of such conditions. Therefore, we used the post-weaning social isolation paradigm, which is an extensively used animal model of childhood neglect (a prevalent form of maltreatment associated with enhanced anxiety [52]) to evaluate the sensitivity of our approach in detecting isolation-induced anxiety. Since the EPM is the most frequently used anxiety test [12], and the LD proved to be the most stable across test repetitions, as well as a strong predictor of future behavior, we reduced our protocol to using these two tests for three baseline and one aversive sampling sessions following isolation. In the EPM, no isolation-related differences were observed on any sampling day when using SiMs (Fig. 3B). However, SuMs were elevated following isolation, requiring only two tests to detect isolation-induced anxiety (Fig. 3C). Since all LD and COMP variables were significantly increased by isolation (Fig. 3E, F, H, I), we also compared their effect sizes across these measures. We found that the effect sizes derived from averaged sampling events (SuMs) were consistently higher than the averages of effect sizes from SiMs, suggesting that SuMs are more sensitive to isolation-induced anxiety than any SiMs (Fig. 3D, G, J). All statistical results for (Fig. 3) are presented in Table 1 In conclusion, the LD and EPM differ in their sensitivity to detect isolation-induced changes in anxiety levels, with SuMs bringing a significant improvement when using the EPM.

#### SuMs trace out quantitatively and qualitatively different gene profiles compared to SiMs

Since SuMs represent robust indicators of anxiety, we next sought to investigate their molecular correlates. To this end, we conducted RNA-sequencing on mPFC (exerting the top-down control of anxiety [30, 31]) tissue of rats in a resting state. Samples from 27 subjects were chosen based on their anxiety levels by ranking their COMP scores in ascending order and selecting every second animal for analysis (Fig. 4A). We found that SuMs outperformed SiMs in the number of identified gene targets, with LD-SuMs and EPM-SuMs increasing discoveries 3-fold and 8-fold, respectively (Fig. 4B). No significant gene expression differences were associated with OF SiMs or SuMs. Interestingly, LD-SiM associated genes largely overlap with LD-SuM, while EPM measures correlate with distinct gene sets (Fig. 4C), suggesting that LD-SuMs offer a similar, but more refined picture about trait anxiety compared to LD-SiMs, whereas EPM-SuMs likely capture distinct processes to EPM-SiMs. Gene-behavior associations were further investigated through the robustness of correlations, which indicated that LD-SiMs, LD-SuMs and EPM-SuMs are also the most stable predictors of behavior, in contrast to the least robust EPM SiMs (Fig. 4F). Surprisingly, the COMP SuM variable only correlated with two gene targets (*Rpl30, Zfp26*), both of which also emerged as correlates of LD SuMs. For information on differentially expressed genes associated with all variables mentioned here, refer to Supplementary Table 1.

Through investigating the functional clusters of genes associated with SiMs or SuMs of different tests, we found that SiMs primarily correlated with metabolic and stress-related genes, while SuMs established a strongly plasticity-focused gene profile (Fig. 4D). The majority of genes associated with SuMs were also putatively involved in cellular localization, cellular transport, potassium signaling, or transcription. Within the plasticity-associated cluster, genes can be further classified into more specific functions, including extracellular matrix shaping (*Adamts4*; *Hapln2*), general modulation of neuronal development (*Fgf14*; *Fat3*; *Fzd3*), transcription (*Btk*; *Tceb2*; *Ascl2*; *Mdm4*) or translation (*Bc1*; *Eifj2*; *Eif2s2*; *Eif3j*; *Upf2*; *Upf3b*). Also, a significant percentage of SuM-associated genes encode direct modulators of neuronal excitability and neurotransmission, such as *Kcnk1*, *Kcnk9*, *Kcna2*, *Kcns2*, and *Kcnj4* (Fig. 4E). These findings suggest that our sampling strategy not only enhances gene discovery quantitatively, but also yields a qualitatively distinct functional gene profile.

To confirm our RNASeq findings with a differently sensitive method, we performed a qPCR analysis on the 43 most robust and the 2 least robust protein-coding genes (Supplementary Table 2). When comparing gene expression levels between the RNASeq and qPCR data, 17.8% (8) of genes showed significant positive correlations (Supplementary Table 3). However, 94.6% of the genes that were not significantly correlated between the two different methods showed a fold change value lower than 1.2 (Fig. 4F, Supplementary Table 1) which is below the typical cut-off criteria for the qPCR analysis [53]. In addition, the remaining 2 non-correlating genes with a fold change higher than 1.2 showed a relatively low expression in the RNASeq and were not detectable with PCR. These were also the 2 least robust genes in our analysis (Supplementary Table 2).

Furthermore, we have examined the differential expression of the aforementioned 45 genes in the amygdala, a key region in the regulation of anxiety, and found that after FDR correction, 15.6% (7) of them showed a significant correlation with anxiety-related variables (SiMs, SuMs, COMPs or behavior in an aversive environment) (Fig. 4G, middle). Additionally, qPCR analysis revealed that 17.8% (8 genes) exhibited significant positive correlations in expression levels between the two areas (Fig. 4G, left). Notably, 6.7% (3 genes) - *Glb1, Pla2g7* and *Shbg* - of the investigated amygdala genes showed significant correlations with both anxiety measures and mPFC gene expression. These findings suggest that some of the gene targets identified by our method may play a broader role in modulating trait anxiety.

#### A consensus score to measure trait anxiety

Based on the above, SuMs offer enhanced predictive power over SiMs as a function of their complexity, e.g. the number of sampling points included. However, each experimental paradigm reaches an inflection point beyond which additional sampling points no longer yield significant improvements in predictive value. To offer a standardised and feasible framework for measuring trait anxiety, we sought to determine a consensus inflection point by comparing the added predictive value of additional tests across all paradigms of our study. In Fig. 5, we show the improvement repeated testing offers in effect sizes related to social isolation, correlations between similar anxiety tests under varying aversive conditions, correlations across different anxiety tests, or correlations between anxiety tests and generalized fear responses alongside molecular predictions from our RNASeq analysis. We expressed the predictive value of different sampling depths (1, 2 or 3 tests) as a percentage of the maximum prediction achieved either within a particular test (Fig. 5B, C) or across all tests (Fig. 5D). We found that the most reliably predictable outcomes were the anxiety-related variables under aversive conditions, i.e. tests under bright light or following social isolation. A single trial of either the EPM or LD test accounted for around 80% of the maximum predictive value in both contexts. However, in the case of anxiety following social isolation, a single EPM trial failed to detect significant behavioral effects. In contrast, the more sensitive EPM-SuMs, and LD-SiM could explain approximately the same variability as multiple LD testing. Predicting outcomes across different contexts proved to be more challenging: significant correlations between anxiety across different tests or with freezing behavior in the safe context of the conditioned fear paradigm generally required multiple test repetitions. Further, identifying molecular correlates of anxiety was the most challenging task, as the number of gene correlates increased progressively with each additional test occasion. The most complex SuM variables captured the highest number of gene targets in both tests. Finally, a comparison of test performance, expressed as correlations relative to the highest prediction achieved across all test variables (Fig. 5D), revealed that the LD test consistently outperformed the EPM across all paradigms. Based on these findings, we provide the optimal testing protocol recommendations in the Discussion.

## Discussion

Our study aimed to model trait anxiety in rodents to enhance the translational potential of preclinical testing. We hypothesized that the frequently used preclinical anxiety tests only measure state anxiety. Since the definition of trait anxiety is the tendency of the subject to respond to situations with elevated state anxiety and avoidance [1, 2], our approach to revealing trait anxiety was to repeat classical anxiety tests multiple times and introduce summary measures of multiple test events and variables.

We present the first experimentally validated behavioral sampling protocol to measure TA in rodent models. Unlike the conventional testing methods using single measure (SiM) behavioral tests, our method uses summary measures (SuMs) from repeated testing, which improve both behavioral and molecular predictions of anxiety tests. While SiMs from the EPM, OF, and LD tests failed to show consistent correlations across tests, SuMs, consisting of multiple variables from repeated tests, revealed significant correlations and were able to uncover the shared driving force of behavior in these tests, TA. We validated SuMs as measures of TA in both male and female rats and mice, finding comparable results across sexes; thus, later experiments focused on males. We showed that SuMs, in proportion with their complexity, enhance inter-test correlations, strengthen predictions of behavior in more threatening environments, and more accurately capture the consequences of chronic stress, when compared to SiMs.

The idea of repeating anxiety tests, especially the EPM, has been controversial because of a phenomenon known as one-trial tolerance. One-trial tolerance refers to two distinct issues; (1) a progressive reduction in exploration in the EPM over repeated testing events, and (2) the decreasing sensitivity of the EPM to detect benzodiazepine effects over repeated testing [54]. Since the EPM was originally developed for assessing benzodiazepine-induced anxiolysis [17], consequently, any experimental condition - such as repeated testing - that diminish the EPM’s ability to detect benzodiazepine effects has traditionally been viewed as compromising the test’s validity. This focus on benzodiazepine responsiveness has, in turn, limited the EPM’s representativity to only a few aspects of trait anxiety. Indeed, there is a long list of conditions and drugs that have no effect on behavior when using the original EPM protocol, but their anxiety-like effects become apparent in different testing conditions, such as more aversive environments, in response to prior stressful experiences, or in a modified EPM [55–57]. We argue that while the carry-over effect is evident in the EPM, valuable additional information can be obtained through multiple testing, as outlined in detail below. Moreover, not all anxiety tests show this carry-over effect, as behavior in the LD test remains stable across repeated trials.

We argue that SuMs capture TA, the shared basis of anxiety-like behavior. This aligns with classical models of Eysenck, Belzung, and the Spielberger state/trait inventory-based clinical practice, which describe SA as a consequence of TA interacting with situational stress [1, 13, 49, 58, 59]. While all these models emphasize that characterizing TA requires repeated sampling across multiple contexts, our approach is the first that sought to apply this principle in preclinical settings. We hypothesized that this translational approach would resolve the inconsistencies previously observed in preclinical models [13, 22, 24]. In our study, increasing variable complexity improved both inter-test correlations and predictive power, suggesting that SuMs reliably converge to TA. To our best knowledge, this is the first preclinical TA paradigm that refines experimental analysis instead of experimental subjects [26–28, 60], enabling detailed individual phenotyping.

We compared SiMs and SuMs of different tests in capturing whole-genome expression profiles in mPFC samples. SuMs revealed both quantitatively and qualitatively distinct gene expression profiles and enhanced gene discoveries up to eightfold compared to SiMs. This likely reflects SuMs’ capacity to explain a greater proportion of variance in gene expression within individuals, allowing the use of high-powered analytical approaches such as continuous anxiety scores over traditional categorical groupings of high- and low-anxiety subpopulations. Furthermore, the enhanced sensitivity of SuMs reduces the number of required subjects, and improves the discovery rate of new gene targets of anxiety [61]. It is important to note, that while the COMP variable identified fewer gene targets than SuMs of EPM or LD did, this result, although somewhat disappointing, is not unexpected. This outcome likely reflects the fact that different anxiety tests revealed largely non-overlapping gene correlates, suggesting that these tests may represent distinct manifestations of anxiety-like behavior. In contrast, SuMs appear to capture more trait-like characteristics, which may explain their broader association with gene expression - though this integrative capacity has its limits. Genes detected with SuMs clustered into functionally different groups than SiMs indicating that besides having superior sensitivity, SuMs explain a previously unrecognized biological variability. These previously undiscovered gene products are potential candidates for the reduction of TA and treatment of anxiety disorders. We also tested the robustness of these molecular findings, further increasing their translational potential.

SuM-associated genes formed functionally interconnected networks tied to neural plasticity and psychiatric disorders [62]. Notably, these included extracellular matrix regulators like *Adamts4* (implicated in perineuronal net remodeling) [62, 63] and *Hapln2*, which stabilizes Ranvier nodes for proper neural conductivity [62]. SuMs were negatively correlated with *Fgf14*, a trophic factor essential for neuronal excitability [64, 65] and positively correlated with several broad modulators of gene transcription and translation, like *Btk*, *Upf2, Upf3b* [66], *Tceb2* [67], *BC1* [68], or *Eif2s2*, *Eifj2*, and *Eif3j* [69, 70]. Finally, we found many differently regulated potassium channels, which are direct modulators of membrane currents. This indicates that trait anxiety correlates with stable alterations in gene expression, excitability, and neuronal plasticity in resting conditions in the mPFC, suggesting novel therapeutic targets focused towards neural circuit adaptability.

We also examined whether transcripts strongly and consistently associated with individual TA in the mPFC also correlated with TA in the amygdala. Although these regions are functionally connected, their molecular profiles differ due to distinct neuronal subtypes and roles in anxiety regulation [30, 71]. The observed 17.8% overlap in TA-associated genes, though modest, is biologically meaningful - reflecting conserved pathways that may underlie cross-regional coordination within anxiety circuits. This shared subset suggests broader molecular shifts across the network, while the >80% divergence underscores region-specific mechanisms. Identifying genes linked to TA in both regions provides a basis for targeting common molecular nodes and developing more effective pharmacological interventions.

To propose a versatile and feasible protocol for measuring TA, we compared the predictive value of SiMs and SuMs of different tests across all paradigms. We compared the performance of the LD and EPM tests in all conditions, representing opposite ends of the robustness spectrum and this way providing a strong framework for understanding how different anxiety measures behave under varying conditions. We found that the predictive strength for behavior or molecular correlates varies across paradigms. While a single LD test was enough to predict behavior in an aversive environment, two were needed for novel contexts, and all three tests were required to reliably link behavior to molecular outcomes. Our findings demonstrated that SuMs were correlated with fear generalization, but not with the acoustic startle response. In humans, high trait anxiety is predictive of fear generalization [72], therefore these findings support our hypothesis that SuMs are able to capture a construct closely related to trait anxiety. Our analysis of behavioral predictions showed that the LD test consistently outperformed the EPM across all paradigms. A single LD test provided similar insight comparable to multiple EPM trials; two LD tests achieved 96% of the maximum predictive power, while SuMs based on three LD trials yielded the strongest molecular associations, identifying four times more anxiety-related genes than any EPM-based variable. Based on these findings, we recommend conducting three LD tests (using time and frequency variables) with one day inter-test intervals to achieve more precise behavioral and molecular predictions. Furthermore, using multiple tests that capture different aspects of anxiety, such as the EPM and LD in combination, can yield a more nuanced understanding of anxiety phenotypes. In summary, here we provide a novel behavioral sampling and analysis pipeline to measure TA in preclinical research. We demonstrate that more complex sampling correlates with deeper phenotypic insight, which was confirmed under various conditions, including baseline and aversive testing environments, multiple different test types, in naïve as well as chronic stress-exposed animals, using different cohorts, and across species and sexes of subjects. Notably, the molecular profiling of TA in female rodents is an important next step to examine the potential sex-specific mPFC differences [73]. Using SuMs in experiments boosts behavioral and molecular phenotyping and predictions, consequently reducing the minimally sufficient sample sizes while maximizing the discovery rate of novel treatment candidates. In addition, SuMs reveal a distinct, plasticity-focused gene profile associated with TA. We encourage the adoption of this refined phenotyping approach, to help bridge the translational gap between preclinical and clinical anxiety research.

## Supplementary information

Supplementary Figures and Tables

Supplementary Materials and Methods

## Data Availability

Raw count values of RNA sequencing data (10.5281/zenodo.14236344) and the corresponding behavioral data (10.5281/zenodo.15469973) is publicly available at the Zenodo online data repository as related datasets. The RNA-seq data is also available in the Gene Expression Omnibus (GEO) under accession number GSE240493.
