# Supplementary material for: Repeated behavioral testing and the use of summary measures reveal trait anxiety in preclinical rodent models
Source: Transl Psychiatry. 2025 Oct 31;15:440. doi: 10.1038/s41398-025-03586-y (PMC12578826; doi:10.1038/s41398-025-03586-y)
Supplement: Supplementary file 1 — Supplementary Figures and Tables [file 41398_2025_3586_MOESM1_ESM.pdf]

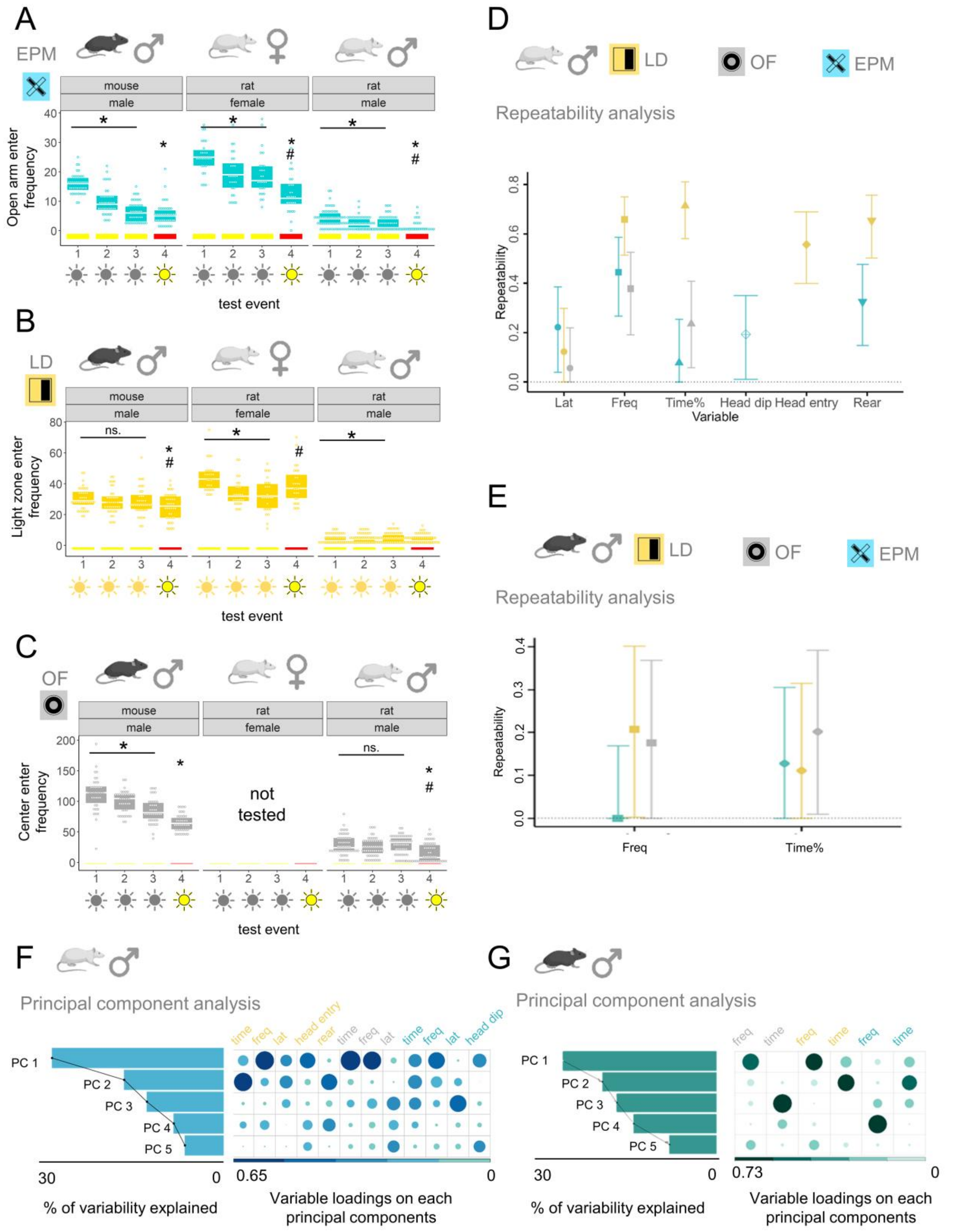

Supplementary Figure 1: Overlaps between anxiety-like variables support the use of SuMs. A-C) Enter frequencies to the aversive compartment of the test in male mice, and female and male Wistar rats in the EPM, LD and OF tests. Yellow and red lines above the x axis represent baseline and aversive conditions, respectively. Sun pictograms indicate light conditions during the test: grey, yellow and bright yellow suns represent no, normal and intense

white light, respectively. Asterisks on lines indicate significant main effect of the test event during the baseline sampling period. Asterisks or hashtags above the aversive sampling day (red line) indicate significant pairwise differences from the 1st or the 3rd test event, respectively. **D-E)** Repeatability scores of variables in different tests in male rats or male mice, respectively. Error bars including the zero line indicate poor repeatability. **F-G)** Principal component analysis of variables collected in male rats and male mice. The percentage of explained variance and variable loadings on each principal component is shown on the left and on the right, respectively. Colors of variable names indicate the test type used.

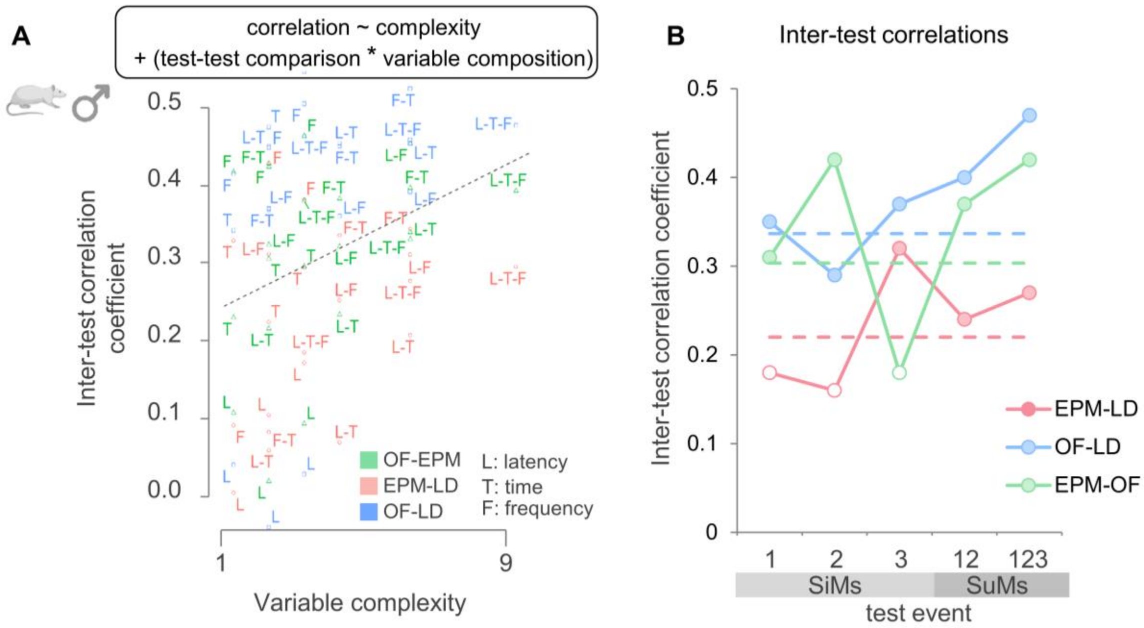

Supplementary Figure 2. Inter-test correlations as a function of variable complexity. A) Inter-test correlations between increasingly complex anxiety variables – SiMs and SuMs – were plotted in response to the number of test types, events, and variables that were included in each measure. According to our final statistical model, inter-test correlations were significantly influenced by variable complexity as a fixed effect, and variable composition and test composition as interacting random effects. The color code indicates different test-test comparisons, while the abbreviations indicate which variables were used to compose a SuM. L: latency, T: time, F: frequency. **B)** Inter-test correlation coefficients shown for all SiM or SuM variables. Here, we show correlations between variables that are used for rats throughout the study based on the results of the repeatability and PCA analyses. SiMs consist of only time spent in aversive zone of a test, while SuMs include time and frequency variables. SiM test events mean the first, second or third test, while SuM 12 means the average of the first two tests, and 123 means the average of all test events. Results from male Wistar rats.

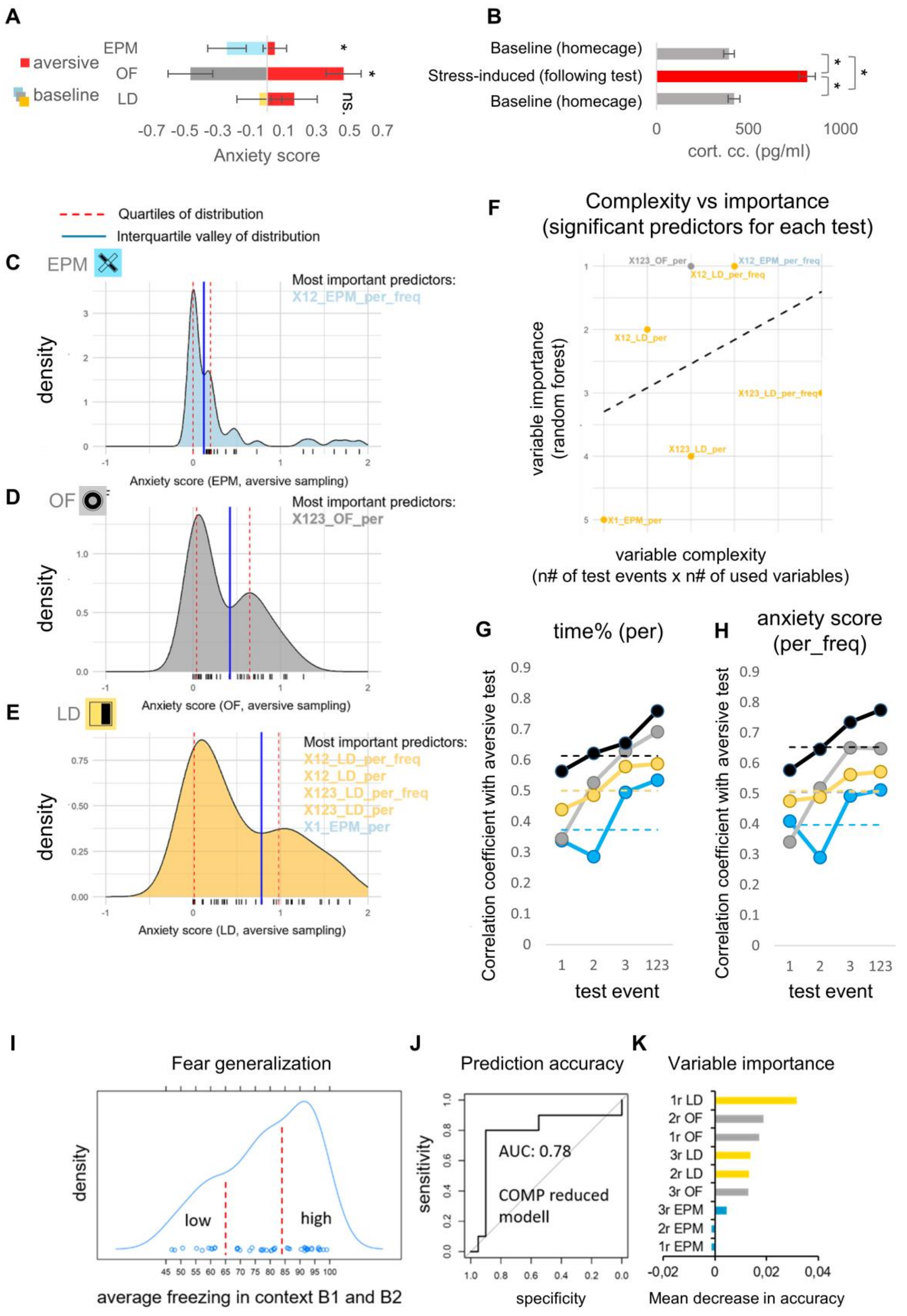

Supplementary Figure 3: SuMs predict acute stress-induced behaviour. A) Anxiety score (average of scaled time% and scaled frequency of entires to the aversive compartment of test)

in the baseline sampling period (no or normal visible light during testing)(left, test-wise colored bars) and in the aversive test event (intense light during testing)(right, red bars) in male Wistar rats. **B)** Corticosterone concentration in baseline (homecage) and stress-induced (following anxiety test) conditions. Upper and lower bars show baseline sampling before and after the whole test-battery, respectively. **C-E)** Density plots of anxiety scores in the aversive test event in the EPM, OF and LD tests, respectively. Data showing a bimodal distribution was separated to low and high anxious groups by finding the lowest density value between lower and upper quartiles (red dashed lines) of the data (interquartile valley of distribution, blue line). Significant baseline sampling period predictors of the anxiety score at the aversive test events are shown in the upper-right corner. Variable names include test events (e.g. X1, X12, X123), test-types (EPM, OF, LD) and variable-types (”per”, ”freq”, or ”per_freq” for time%, frequency or their combination, respectively). **F)** Variable importance in the random forest model was plotted against the complexity of significant predictor variables for anxiety scores in the aversive test event. **G-H)** Correlations between significant predictor-types (time% or anxiety score) of the baseline test events and the anxiety scores of the aversive test event. Note that SuM variables (123) are higher compared to most SiM variables. **I-K)** Density plot, prediction accuracy and variable importance of the random forest model to predict fear generalization in a fear conditioning paradigm.

model	samp-ling	gene symbol	base means	$\log_2$ FC	$\log_2$ FC SE	robust-ness	FDR-adjusted p-value	IHW weight
EPM	SiM	Cytip	23.606	4.028	0.960	3	0.050	2.771
		F7	22.638	4.342	1.022	3	0.050	2.771
		Mrc1	155.711	3.277	0.780	5	0.050	4.962
	SuM	Anp32a	1803.473	0.481	0.113	9	0.013	2.500
		Bc1	25643.881	3.720	0.568	14	0.000	2.447
		Btk	38.921	2.995	0.763	8	0.045	1.325
		Ccdc112	176.938	0.929	0.218	9	0.026	0.650
		Cfdp1	806.939	0.499	0.126	10	0.026	2.500
		Cir1	476.013	0.870	0.191	8	0.004	2.500
		Dusp7	851.688	-0.534	0.130	6	0.016	3.026
		Eif2s2	1070.662	0.767	0.205	5	0.045	2.500
		Eif3j	696.432	1.062	0.205	13	0.001	1.200

		Fam50a	167.672	1.704	0.434	7	0.045	1.200
		Hmgn5	110.513	1.294	0.299	9	0.022	0.673
		Kcnj4	908.471	-0.407	0.110	11	0.045	3.026
		Krcc1	239.564	0.999	0.230	10	0.021	0.673
		Larp7	525.165	0.753	0.162	9	0.003	2.500
		Map7d2	1219.487	0.674	0.137	8	0.001	2.500
		Map9	582.845	1.049	0.270	6	0.033	2.500
		Mphosph8	1155.360	0.727	0.157	8	0.003	2.500
		Nkap	140.282	1.109	0.257	7	0.016	1.200
		Nsrp1	294.536	0.949	0.217	7	0.020	0.627
		Ppig	1241.829	1.107	0.196	10	0.000	2.500
		Sec62	1834.781	1.166	0.207	12	0.000	2.447
		Taf3	336.897	0.595	0.143	9	0.035	0.673
		Top1	420.345	0.825	0.192	7	0.017	1.200
		Upf2	529.868	0.623	0.142	7	0.008	2.500
		Upf3b	574.895	0.998	0.246	8	0.046	0.627
LD	SiM	Abhd1	145.172	0.460	0.112	13	0.034	0.999
		Adamts4	78.607	0.652	0.165	9	0.049	1.288
		Blcap	1577.023	0.291	0.065	11	0.024	0.449
		Col5a3	326.879	0.586	0.149	8	0.026	4.378
		Dgcr6	933.387	0.385	0.093	11	0.033	1.062
		Glb1	160.858	0.857	0.153	15	0.000	1.288
		Gpx3	181.445	0.983	0.234	9	0.024	1.464
		Hapln2	334.366	1.182	0.288	7	0.031	1.288
		Inpp5f	699.704	0.417	0.095	10	0.019	1.046
		Nat6	285.529	0.393	0.092	11	0.024	1.288
		Orai1	104.910	0.963	0.225	10	0.026	0.789

	Pdlim4	397.688	0.410	0.107	9	0.031	4.313
	Pla2g7	184.910	-0.932	0.209	10	0.018	0.999
	Plekhh1	406.534	0.988	0.235	8	0.015	4.378
	Rmrp	40.122	-6.810	1.370	0	0.004	1.288
	Rnf145	1136.341	-0.283	0.059	13	0.004	1.244
	Sbno1	1076.216	-0.406	0.081	14	0.004	0.825
	Shbg	53.145	1.004	0.207	13	0.004	1.252
	Zbed5	428.279	0.362	0.099	8	0.048	4.313
SuM	Adamts4	78.607	0.570	0.159	7	0.047	1.587
	Agpat5	483.012	-0.263	0.070	6	0.035	1.652
	Alg10	32.986	-1.880	0.460	7	0.015	1.652
	Ascl2	66.192	1.032	0.245	6	0.013	1.959
	Bhlhe40	1299.835	-0.301	0.080	6	0.029	2.396
	Blcap	1577.023	0.291	0.057	12	0.004	0.640
	Blvra	296.247	-0.473	0.133	4	0.044	2.044
	Ccnjl	34.154	0.969	0.225	9	0.011	1.652
	Cdc42ep2	202.890	0.374	0.095	10	0.023	1.652
	Cdyl2	59.969	-0.790	0.210	6	0.034	1.959
	Cnih2	2437.705	0.352	0.076	10	0.004	2.396
	Col5a3	326.879	0.634	0.127	12	0.004	1.448
	Dctd	48.110	0.596	0.166	8	0.047	1.652
	Dgcr6	933.387	0.361	0.087	10	0.021	0.847
	Dnaaf5	117.892	0.461	0.128	8	0.046	1.476
	Dnajb14	67.919	-1.098	0.293	4	0.042	1.120
	Dnpep	964.720	0.254	0.067	8	0.044	0.913
	Dpm2	315.731	0.369	0.102	6	0.045	1.589
	Eral1	351.824	-0.308	0.084	9	0.037	2.044

	Evi2a	277.589	0.618	0.168	4	0.042	1.448
	Fah	200.247	0.497	0.122	6	0.015	1.652
	Fam122a	214.924	0.348	0.088	12	0.024	1.549
	Fat3	152.133	-1.018	0.277	6	0.042	1.476
	Fbxw5	737.113	0.287	0.070	8	0.037	0.347
	Fem1b	221.242	-0.608	0.172	4	0.049	1.698
	Fgf14	31.569	-0.802	0.221	8	0.042	1.834
	Fzd3	55.025	-1.216	0.277	6	0.009	1.959
	Glb1	160.858	0.699	0.160	10	0.010	1.698
	Gpx3	181.445	0.974	0.213	9	0.006	1.652
	Hapln2	334.366	1.080	0.272	5	0.023	1.448
	Kcna2	36.710	-2.040	0.545	4	0.035	1.959
	Kcnk1	1531.718	-0.273	0.077	5	0.042	2.396
	Kcnk9	52.273	-1.220	0.334	8	0.042	1.834
	Kcns2	60.124	-0.745	0.180	9	0.014	1.959
	Kit	183.256	-0.620	0.148	9	0.014	1.652
	Klhl28	38.999	-1.140	0.323	5	0.048	1.834
	Lcn12	37.886	0.785	0.208	8	0.035	1.652
	Lemd3	277.339	-0.322	0.088	6	0.042	1.589
	Lrp1b	352.926	-0.844	0.236	4	0.049	1.409
	Lyrm1	182.250	0.463	0.126	7	0.042	1.698
	Mdm4	95.155	-1.173	0.319	4	0.042	1.587
	Mllt4	1188.392	-0.247	0.065	8	0.042	0.868
	Nat6	285.529	0.341	0.089	8	0.027	2.044
	Orai1	104.910	0.908	0.212	9	0.013	1.476
	Pds5a	192.446	-0.572	0.156	6	0.042	1.549
	Pla2g7	184.910	-0.807	0.205	8	0.024	1.549

	Pvrl2	86.300	0.495	0.132	10	0.037	1.597
	Rab26	1010.801	0.305	0.081	8	0.044	0.847
	RGD1559904	298.985	-0.327	0.091	7	0.045	1.652
	Rnf145	1136.341	-0.268	0.054	11	0.004	0.347
	Rpl30	472.323	0.662	0.164	7	0.019	1.507
	Rragd	188.492	-0.452	0.107	9	0.013	1.698
	Samd4b	829.421	0.215	0.051	12	0.029	0.347
	Sbno1	1076.216	-0.347	0.081	10	0.014	0.847
	Scand1	644.680	0.484	0.115	9	0.014	1.589
	Shbg	53.145	0.942	0.195	12	0.004	1.652
	Slc25a10	322.184	0.392	0.095	9	0.015	1.448
	Slc25a28	536.981	0.295	0.071	8	0.015	1.507
	Smcr8	96.412	-0.931	0.227	8	0.015	1.658
	Snta1	641.738	0.223	0.062	8	0.047	1.467
	Sts	173.874	0.531	0.147	6	0.044	1.698
	Tatdn3	213.583	0.528	0.113	10	0.004	1.698
	Tceb2	829.548	0.475	0.112	8	0.015	0.913
	Tmem164	542.956	-0.220	0.061	10	0.045	1.652
	Tmem196	166.953	-0.713	0.188	7	0.029	2.044
	Tmx2	1251.797	-0.286	0.077	5	0.048	0.868
	Tspan1	49.982	0.777	0.206	7	0.042	1.120
	Ttbk2	69.348	-1.574	0.349	6	0.009	1.120
	Ttc39b	71.332	-0.981	0.256	5	0.027	1.959
	Ttc9	521.426	-0.276	0.073	6	0.035	1.652
	Vcpip1	199.177	-0.799	0.222	5	0.045	1.652
	Wdr34	166.398	0.350	0.098	7	0.047	1.549

		Xkr6	81.662	0.814	0.193	8	0.013	1.587
		Zfp26	155.941	-0.483	0.109	10	0.009	1.658
		Zyx	607.518	0.409	0.095	9	0.011	1.589
COMP	SuM	Rpl30	472.323	0.597	0.130	Not calculated	0.0464	1
		Zfp26	155.941	-0.407	0.090	Not calculated	0.0464	1.097

Supplementary Table 1. Parameters of significant RNA expression – behavior associations sorted by different sampling and analysis approaches. **Abbreviations:** log2FC: log2 fold-change, SE: standard error, FDR: false discovery rate, IHW: independent hypothesis weighting. Results from male Wistar rats.

gene	robustness
Cytip	3
F7	3
Hapln2	7
Rragd	9
Scand1	9
Zyx	9
Anp32a	9
Kit	9
Larp7	9
Ccnjl	9
Gpx3	9
Eral1	9

Pdlim4	9
Hmgn5	9
Ccdc112	9
Kcns2	9
Slc25a10	9
Taf3	9
Adamts4	9
Inpp5f	10
Pvrl2	10
Tmem164	10
Zfp26	10
Cfdp1	10
Orai1	10
Tatdn3	10
Cnih2	10
Ppig	10
Pla2g7	10
Krcc1	10
Cdc42ep2	10
Dgcr6	11
Kcnj4	11
Nat6	11
Blcap	12
Samd4b	12
Col5a3	12

Fam122a	12
Sec62	12
Rnf145	13
Abhd1	13
Shbg	13
Eif3j	13
Sbno1	14
Glb1	15

Supplementary Table 2. Genes analyzed via qPCR in the amygdala, chosen for their robustness scores in the RNASeq analysis of mPFC tissue. Results from male Wistar rats.

comparisons	gene symbol	r	p	significance after FDR correction	correlating variables
PFC PCR vs RNASeq correlations	Adamts4	0.533578	4.15E-03	*	-
	Col5a3	0.559829	2.39E-03	*	-
	Glb1	0.608059	7.66E-04	*	-
	Gpx3	0.580586	1.50E-03	*	-
	Hapln2	0.741758	9.52E-06	*	-
	Pdlim4	0.458861	1.61E-02	*	-
	Pla2g7	0.568376	1.98E-03	*	-

	Tatdn3	0.615385	6.34E-04	*	-
AMY vs PFC expression level correlations	Glb1	0.647741	0.000259	*	-
	Larp7	0.59655	0.001022	*	-
	Pla2g7	0.574481	0.001725	*	-
	Kcns2	0.482295	0.010843	*	-
	Hmgn5b	0.47779	0.011718	*	-
	Tatdn3	0.473748	0.012553	*	-
	Mrc1	0.407814	0.034722	*	-
	Shbg	0.397436	0.040087	*	-
AMY correlations with SiMs, SuMs, COMPs or aversive behavior	Blcap	0.40116	0.038091	*	EPM SuM
	Glb1	0.605006	0.000828	*	LD SiM
	Glb1	0.394383	0.041784	*	COMP SuM
	Inpp5f	-0.39178	0.043279	*	EPM aversive
	Pla2g7	-0.58669	0.001297	*	LD SiM
	Pvrl2	0.388032	0.045499	*	EPM SiM
	Rragd	-0.39769	0.048988	*	OF aversive
	Shbg	0.398459	0.048511	*	OF aversive
	Abhd1	-0.4359	0.023036	ns.	COMP SuM
	Abhd1	-0.40842	0.034426	ns.	EPM SuM
	Abhd1	-0.40574	0.035746	ns.	EPM SiM
	Anp32a	0.515873	0.005881	ns.	EPM SuM
	Ccnjl	-0.44375	0.020418	ns.	EPM aversive
	Cfdp1	-0.42125	0.02865	ns.	LD SiM
	Cnih2	0.47619	0.012043	ns.	EPM SuM
	Cnih2	0.448176	0.019053	ns.	EPM SiM
	Glb1	0.47558	0.012169	ns.	LD SuM
	Gpx3	0.465201	0.014481	ns.	EPM SuM

	Gpx3	0.434066	0.023684	ns.	LD aversive
	Kit	-0.49112	0.009286	ns.	EPM aversive
	Larp7	-0.42467	0.027248	ns.	EPM aversive
	Orai1	-0.49611	0.008492	ns.	EPM SiM
	Orai1	-0.44505	0.020007	ns.	EPM SuM
	Orai1	0.426129	0.026668	ns.	LD SiM
	Pla2g7	-0.51709	0.005745	ns.	LD SuM
	Ppig	-0.43535	0.023227	ns.	LD SiM
	Rnf145	-0.42467	0.027248	ns.	EPM aversive
	Samd4b	0.470274	0.017678	ns.	OF aversive
	Samd4b	0.384005	0.047986	ns.	EPM SuM
	Scand1	-0.43224	0.024347	ns.	EPM aversive
	Tmem164	0.489391	0.009575	ns.	EPM SiM
	Tmem164	0.470696	0.013215	ns.	EPM SuM
	Tmem164	0.407814	0.034722	ns.	LD aversive
	Tmem164	0.400003	0.047566	ns.	OF aversive
	Zfp26	-0.40296	0.037155	ns.	EPM aversive
	Zyx	0.42735	0.02619	ns.	LD aversive

Supplementary Table 3. Validation of RNASeq experiments, correlations between amygdala expression and mPFC expression, or amygdala expression and anxiety. Results from male Wistar rats. * significant after false discovery rate (FDR) correction. Ns. – not significant after FDR correction
