# Supplementary material for: Repeated behavioral testing and the use of summary measures reveal trait anxiety in preclinical rodent models
Source: Transl Psychiatry. 2025 Oct 31;15:440. doi: 10.1038/s41398-025-03586-y (PMC12578826; doi:10.1038/s41398-025-03586-y)
Supplement: Supplementary file 2 — Supplementary Materials and Methods [file 41398_2025_3586_MOESM2_ESM.docx]

**SUPPLEMENTARY MATERIALS AND METHODS**

**Animals and housing.** Animals were kept in standard environmental conditions of 22±1°C temperature and 60±10% relative humidity in a 12:12h reversed circadian light-cycle room, with lights off at 8.00 a.m. Animals were housed in groups of 2-5 (mice and rats in separate rooms), rats in 1291H Eurostandard type III H cages (42.5x26.6x18.5 cm), and mice in 1284L EUROSTANDARD TYPE II L cages (36.5x20.7x14 cm). Water and food (Sniff, Soest, Germany) were available *ad libitum*. Experimental subjects underwent a 2-week acclimatisation period to their housing room, and also three 1-minute sessions of handling by the experimenter on three consecutive days right before experimentation. To identify individual subjects, their tails were marked with a black marker. Identification marks were first applied during the handling period and renewed every three days. All experiments were approved by the Animal Welfare Committee of the Institute of Experimental Medicine, the National Scientific Ethical Committee on Animal Experimentation (permit no. PE/EA/00220-4/2022) and are in agreement with the 2010/63/EU directive.

**Supplementary Materials Table 1.: Overview of tests conducted in each animal cohort.**

Two cohorts of adult male Wistar rats (Charles-River, n=54, age= 100 days) were used to examine trait anxiety and its neural correlates. To assess replicability and potential sex differences, behavioral assessment was repeated with adult female Wistar rats (Charles-River, n=27, age= 130 days), and adult male C57BL/6J mice (Jackson Laboratory, n=40, age= 95 days). One cohort of adult male Long-Evans rats (Charles-River, n=27, age= 110 days) was used to explore the correlations between acoustic startle responses, generalized fear in the conditioned fear paradigm (CFP), and trait anxiety. Additionally, one cohort of adult male Wistar rats (Charles-River, n=30, age at testing= 180 days) was used to investigate the effects of social isolation on trait anxiety. Sample sizes for the first rat experiment (n=54) were calculated using the pwr R package aiming to measure a minimum r=0.35 spearman linear correlation coefficient at alpha=0.05 significance level and beta=0.8 power. Following the first experiment we decreased sample sizes according to the robustness indices of behavior-behavior and behavior-gene expression correlations.

**Experimental design.** Wistar rats were tested according to a semi-randomized testing protocol for 3+2 weeks (Fig.1). The three-week test battery involved the EPM, OF, and LD tests, each repeated three times for all individuals. The semi-randomised testing order involved six fixed combinations of tests with balanced sample sizes. Afterwards, an additional week of testing was conducted under more aversive conditions with increased light intensities. Two days after the aversive week, a different type of open field test (OF2) was performed as the last test for all animals, so that their last test experience before termination was identical. For the social isolation protocol, upon weaning at P21, animals were reared in groups of 4 (social), or alone in a cage (isolated) until testing in adulthood. Female rats, male rats in the social isolation cohort and mice underwent the 3-week baseline anxiety testing, and the additional week of testing in aversive conditions. Long-Evans rats first underwent the above described 3-week anxiety testing protocol, followed by an acoustic startle paradigm 3 days later, and finally a fear conditioning paradigm a week after that. For the experiments described above, animals were randomly chosen for each group. For anxiety tests, time spent in the aversive zone of tests, frequency, and latency of aversive zone entries were analyzed automatically using Noldus EthoVision XT15, head-dips in EPM and head-entry for the LD, as well as rearing for both tests were analyzed manually using Solomon Coder by experimenters that were blind to experimental groups.

**Behavioural tests.** All behavioural experiments were carried out in the first half of the animals’ active phase by the same experimenter, in a testing room adjacent to the housing room. Animals were individually transferred from their homecage to the experimental room in a transfer-cage. Following the last behavioural test, animals were left undisturbed for 2 weeks, then swiftly terminated by decapitation in a non-stressed baseline state for later analysis of neurobiological correlates described below.

The Elevated Plus-Maze test consisted of an elevated plus-shaped testing arena with black plexiglass walls and a white plexiglass floor. The apparatus consisted of two open (aversive) and two enclosed (protected) arms connected by a central area (rat: height 70cm, arms 42x12 cm, centre 12x12 cm, closed arm wall height 35 cm; mouse: height 50cm, arms 30x7 cm, centre 7x7 cm, closed arm wall height 30 cm). Animals were placed in the centre of the testing arena facing the open arm in infrared lighting (13 lux), and were recorded for 5 minutes for later analysis. Aversive sampling was done in dim white lighting (49 lux).

The Light-Dark test consisted of an open, dimly lit (49 lux white light) arena with transparent plexiglass walls, and a closed, dark arena with black plexiglass walls and a black cover. The two chambers were connected by a narrow door (rat: light and dark boxes 50x50x40 cm; mouse: light and dark boxes 40x20x25 cm). Animals were placed in the light compartment and were recorded for 10 minutes. Aversive sampling was done in bright lighting (422 lux).

The Open Field test consisted of a large circular arena with black metal walls and a black wooden floor (for rats, diameter 100 cm, height 40 cm), or a white plastic box (for mice, 40x30x15 cm). The inner 50% (for mice) or 70% (for rats) of the arena was appointed as the centre. Animals were placed in the periphery of the arena in infra-red lighting, and were recorded for 10 minutes. Aversive sampling was done in the same experimental apparatus, but in dim white lighting (49 lux).

The different type of Open Field testing (OF2) was done in a different experimental room by a different experimenter in dim white light. The testing apparatus was a black plastic square-shaped box (79x54x35 cm). Animals were placed in the periphery of the arena and recorded for 5 minutes.

The acoustic startle paradigm was performed with Long-Evans rats by a different experimenter 3 days after their last anxiety test. Startle reactivity was assessed in Plexiglas chambers (length: 25 cm, diameter: 12.5 cm) placed in sound-attenuating boxes (33 x 33 x 48 cm) controlled by the SR-LAB software (SR-LAB Startle Response System, San Diego Instruments, USA) as previously described^1^. Briefly, startle reactivity for increasing pulse intensity was assessed during a single session (approx. 20 min, light on in chambers). Session started with the delivery of 5 each of 120 dB startle pulses (over 65 dB background white noise) allowing startle to reach a stable level before specific testing. In a second block we presented four of each startle stimulus intensities (80, 90, 100, 110, and 120 dB) in a pseudorandom order with an average 15 sec inter-trial intervals (range of 7-23 sec) between stimulus presentations. The average startle response for each intensity was calculated and considered as the index of startle reactivity.

The fear conditioning paradigm was carried out with Long-Evans rats 1 week after the acoustic startle paradigm (Fig.2F-H). Fear conditioning was conducted in a different, brightly lit experimental room. Rats were placed in a 30x30x30 cm plexiglass chamber with metal grid floors. After a 2.5 min habituation period, 2.4 mA, 1 sec long, inescapable electric foot-shocks were delivered through the grid floor 10 times, each with a 30 sec inter-shock interval. Animals were placed in the same chamber 28 days later as a contextual reminder, then tested for fear-generalisation in a different apparatus with different contextual cues on the following day. Measured behavioural outcome was time spent freezing. Freezing was quantified by Ethovision as immobility lasting longer than 1 second, with detection thresholds and settings validated by significant correlation with expert hand-scoring (r>0.9).

For the social isolation protocol, upon weaning at P21, animals were reared in groups of 4 (social), or alone in a cage (isolated) for approx. 5 months until testing in adulthood.

**Statistical analysis of behaviour.** All statistical analysis was done in R statistical environment (version 3.6.2.)^2^, through its integrated development environment, RStudio (version 1.3.1056)^3^.

| **variable** | **species** | **summarisation** | **variable types** | **test events** | **test types** |
| --- | --- | --- | --- | --- | --- |
| **SiM** | rat | - | time | 1 | EPM/OF/LD |
| **SuM** |  | averaging | time, frequency | 2 or 3 | EPM/OF/LD |
| **COMP** |  |  | time, frequency | 1 or 2 or 3 | EPM+OF+LD |
| **SiM** | mouse | - | frequency | 1 | EPM/OF/LD |
| **SuM** |  | averaging | frequency | 2 or 3 | EPM/OF/LD |
| **COMP** |  |  | frequency | 1 or 2 or 3 | EPM+OF+LD |

**Supplementary Methods Table 2. Composition of SiMs, SuMs or COMPs.**

**Blood corticosterone analysis of Wistar rats.** Tail vein blood samples (0.3-0.5 ml) were collected in a resting state (5 days before the first experiment) and in stress-induced (immediately after OF2) conditions, and trunk blood was collected into ice-cold EDTA-containing tubes at the time of termination in a baseline state. After sampling, blood-containing tubes were centrifuged at 4 °C, and plasma was separated and stored at -20 °C until analysis. The quantification of plasma corticosterone was carried out using radioimmunoassay similarly to previous work by our laboratory^4^. Briefly, corticosterone was separated from corticosteroid-binding globulin (CBG) at low pH levels. CBG was kept inactive at low pH levels to avoid interference. Rabbit antiserum against corticosterone-3-carboxymethyloxime-bovine serum albumin was developed in the Institute, and 125I-labeled carboxymethyloxime–tyrosine–methyl ester was used as the tracer. All samples were measured in the same assay, with a sensitivity of 1 pmol/ml.

**RNA Sequencing of mPFC tissue.** Animals were swiftly decapitated after being transferred directly from their home-cage, their brains were removed, then cooled and washed in ice-cold saline. A 1 mm coronal section between Bregma 3.2-2.2 mm was sliced in a cooled slice matrix. Bilateral medial prefrontal cortex samples were dissected on ice as defined by the forceps minor of corpus callosum and the medial wall of the hemisphere (i.e. including the infralimbic, prelimbic and anterior cingulate cortices). Samples were immediately placed in Eppendorf tubes on dry ice and stored at -80C temperature until RNA isolation. Whole transcriptome-analysis via RNASeq was performed on the homogenised mPFC tissue. RNA sequencing was performed on 27 animals, chosen by their COMP scores. More precisely, a scale was created from all 54 animals’ SuM scores, and every second animal in the scale was selected for RNASeq analysis. After the selection, correlations between the baseline composite score and behaviour in aversive conditions remained significant for both the selected and non-selected groups (Fig.4A). Analysis of gene-behaviour regressions was carried out using a negative binomial generalised linear model, with anxiety-scales generated from behaviour in different test-types (EPM/OF/LD/COMP) across test repetitions (SiM/SuM) as continuous covariates in the design. This was done to avoid losing relevant behavioural information by binarising the population into “high-anxiety” or “low-anxiety” groups. Percent of time spent in the aversive zone was used for SiMs, and composite scores of time and entry frequency were used for SuMs.

**Robustness.** We characterised gene-behaviour associations with the per unit of RNA read count change of an anxiety measure (SiM or SuM) and robustness. A robustness index estimates the smallest number of randomly excludable samples that likely compromise the statistical significance of a given correlation. The index was calculated by a custom-written script that reanalyses the Spearman correlations across randomly selected, sub-cohorts of varying sizes from the original population. A total of 20.000 correlations were computed across sub-cohorts of all possible sample sizes, and minimums, maximums, standard deviations and means of their p-values were calculated. Robustness index was defined as the smallest sub-cohort size in which the standard deviation of the correlations' p-values reached the 0.05 alpha threshold level. The R scripts containing functions and calculations used are available upon request.

**qPCR analysis.** Total RNA was isolated using the RNeasy Lipid Tissue Mini Kit (Qiagen). The quality and quantity of the isolated RNA were assessed using the Qubit RNA BR Assay Kit and Qubit RNA IQ Assay Kit (Invitrogen, USA). A total of 500 ng of RNA was used for reverse transcription, which was performed using the High Capacity cDNA Reverse Transcription Kit (Thermo Fisher Scientific, USA). The concentrations of the resulting cDNA were determined using the Qubit ssDNA Assay Kit (Invitrogen, USA). Gene expression patterns were analyzed using custom 384-well TaqMan Gene Expression Array Cards (Applied Biosystems, USA). Each array card ran eight samples, including seven test samples and one run-control sample for calibration. The cDNA samples (200 ng each) were diluted and mixed with the TaqMan Gene Expression Master Mix to create a qPCR reaction mix, with a final concentration of 1 ng/µl. The qPCR reactions were performed using the ViiA 7 Real-Time PCR System (Applied Biosystems, USA), and the data were collected using the QuantStudio software (Applied Biosystems, USA).

We performed qPCR analysis of total RNA from the mPFC and amygdala (AMY) samples of the same animals used for RNASeq. For mPFC, the RNA sample isolated for RNASeq was used for further analysis. Bilateral microdissection of AMY (including MeA, CeA, BLA and BMA, between Bregma -2.0 and -3.0, defined by the external and internal capsules and optic tract) was performed as described for mPFC tissue. Gene expression patterns were analyzed using custom 384-well TaqMan Gene Expression Array Cards (Applied Biosystems, USA). These cards measured the expression of 45 genes of interest, which were selected based on the robustness of mPFC RNA sequencing experiments. Inter-run calibration values were calculated based on the run control samples, using Actb and Gapdh as reference genes, as per the method described by Hellemans. The normalized relative gene expression values were calculated using the 2-ΔΔCT method. These values were further normalized to the inter-run calibration values. Expression values with threshold cycles greater than 35 were excluded from the analyses.

Total RNA was isolated from mPFC and amygdala (AMY) samples of the same animals used for RNASeq, using the RNeasy Lipid Tissue Mini Kit (Qiagen), with bilateral microdissection performed for both regions. RNA quality and quantity were assessed using the Qubit RNA BR and IQ Assay Kits (Invitrogen, USA). For cDNA synthesis, 500 ng of RNA was reverse transcribed using the High Capacity cDNA Reverse Transcription Kit (Thermo Fisher Scientific, USA), and cDNA concentrations were measured with the Qubit ssDNA Assay Kit. Gene expression patterns were analyzed using custom 384-well TaqMan Gene Expression Array Cards (Applied Biosystems, USA), which measured 45 genes selected based on mPFC RNASeq results; each card included seven test samples and one run-control for calibration. cDNA samples (200 ng each) were diluted and mixed with TaqMan Gene Expression Master Mix to a final concentration of 1 ng/µl, and qPCR reactions were run on the ViiA 7 Real-Time PCR System with data collected using QuantStudio software. Inter-run calibration values were calculated using Actb and Gapdh as reference genes, following Hellemans’ method. Normalized relative gene expression values were calculated using the 2-ΔΔCT method and further normalized to inter-run calibration values, with expression values above a threshold cycle of 35 excluded from analysis.

**Bibliography**

1. Toth, M., Gresack, J. E., Bangasser, D. A., Plona, Z., Valentino, R. J., Flandreau, E. I., *et al.* Forebrain-Specific CRF Overproduction During Development is Sufficient to Induce Enduring Anxiety and Startle Abnormalities in Adult Mice. *Neuropsychopharmacol* **39**, 1409–1419 (2014).

2. RCoreTeam. R: A language and environment for statistical computing. R Foundation for Statistical Computing, Vienna, Austria. (2019).

3. RStudio Team. RStudio: Integrated Development Environment for R. (2020).

4. Toth, M., Mikics, E., Tulogdi, A., Aliczki, M. & Haller, J. Post-weaning social isolation induces abnormal forms of aggression in conjunction with increased glucocorticoid and autonomic stress responses. *Hormones and Behavior* **60**, 28–36 (2011).
